# Filter-Aided Extracellular Vesicle Enrichment (FAEVEr) for Proteomics

**DOI:** 10.1016/j.mcpro.2025.100907

**Published:** 2025-01-21

**Authors:** Jarne Pauwels, Tessa Van de Steene, Jana Van de Velde, Freya De Muyer, Danaë De Pauw, Femke Baeke, Sven Eyckerman, Kris Gevaert

**Affiliations:** 1 VIB-UGent Center for Medical Biotechnology, VIB, Ghent, Belgium; 2 Department of Biomolecular Medicine, Ghent University, Ghent, Belgium; 3 Ghent University Expertise Center for Transmission Electron Microscopy and VIB BioImaging Core, Ghent, Belgium; 4 Department of Biomedical Molecular Biology, Ghent University, VIB Center for Inflammation Research, Ghent, Belgium

**Keywords:** extracellular vesicles, 300 kDa MWCO ultrafiltration, conditioned medium, starvation, EV-depleted serum, Tween-20, LC-MS/MS, proteomics

## Abstract

Extracellular vesicles (EVs), membrane-delimited nanovesicles that are secreted by cells into the extracellular environment, are gaining substantial interest due to their involvement in cellular homeostasis and their contribution to disease pathology. The latter in particular has led to an exponential increase in interest in EVs as they are considered to be circulating packages containing potential biomarkers and are also a possible biological means to deliver drugs in a cell-specific manner. However, several challenges hamper straightforward proteome analysis of EVs as they are generally low abundant and reside in complex biological matrices. These matrices typically contain abundant proteins at concentrations that vastly exceed the concentrations of proteins found in the EV proteome. Therefore, extensive EV isolation and purification protocols are imperative and many have been developed, including (density) ultracentrifugation, size-exclusion, and precipitation methods. Here, we describe filter-aided extracellular vesicle enrichment (FAEVEr) as an approach based on 300 kDa molecular weight cutoff filtration that allows the processing of multiple samples in parallel within a reasonable time frame and at moderate cost. We demonstrate that FAEVEr is capable of quantitatively retaining EV particles on filters, while allowing extensive washing with the mild detergent Tween-20 to remove interfering non-EV proteins. The retained particles are directly lysed on the filter for a complete recovery of the EV protein cargo toward proteome analysis. Here, we validate and optimize FAEVEr on recombinant EV material and apply it on conditioned medium as well as on complex bovine serum, human plasma, and urine. Our results indicate that EVs isolated from MCF7 cells cultured with or without serum have a drastic different proteome because of nutrient deprivation.

## Highlights

• Efficient enrichment of extracellular vesicles by 300 kDa MWCO ultrafiltration.
• Tween-20 dramatically increases purity, yet keeps extracellular vesicles intact.
• Streamlined workflow for reproducible proteomics with minimal sample loss.
• Promising potential for large-scale comparative studies.
• Serum deprivation dramatically changes the proteome of extracellular vesicles.

## Graphical Abstract

**Figure:**
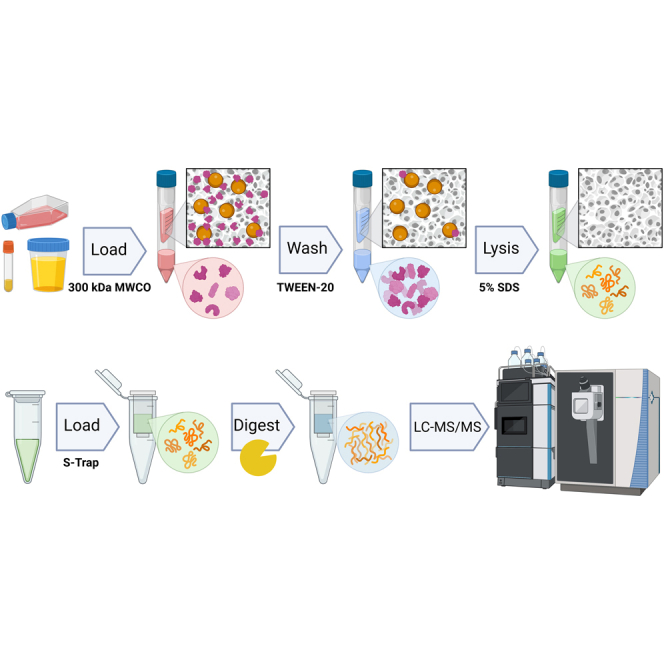

## In Brief

Isolating extracellular vesicles from a complex background proves challenging, especially in a cost- and time-effective high-throughput setting. Here, a simple dead-end ultrafiltration strategy is proposed that uses the detergent Tween-20 to dislodge nonspecific protein adherence to the filter membrane. Using LC-MS/MS analysis, we show a dramatic decrease of contaminant proteins that allow reproducible deep proteomics of the purified vesicles from different sources within a limited time frame using standard lab equipment.

Extracellular vesicles (EVs) make up a heterogeneous population of membrane-enclosed particles that are released by virtually all cells into their extracellular (EC) environment and in bodily fluids, such as plasma, urine, and cerebrospinal fluid. In general, EVs are subdivided in three main categories according to their biogenesis and size (1). The smallest (30–150 nm), termed exosomes, are generated by invaginations at the endosomal membrane, in a transport (endosomal sorting complexes required for transport, ESCRT) dependent (2, 3) or independent manner (4). During this process, early endosomes mature into a multivesicular body (MVB) that holds multiple membrane delineated vesicles termed intraluminal vesicles (ILVs). Once the MVB fuses with the plasma membrane (PM), these ILVs are released into the EC environment as exosomes. Microvesicles (100–500 nm) are formed by an immediate outward budding of the PM, orchestrated by the ESCRT machinery (5). These protrusions are ultimately pinched off and released from the cell. Finally, apoptotic bodies (50–5000 nm) are formed during apoptotic cell disassembly with large extrusions at the PM, fragmenting dying cells in large blobs that may even contain organelles (6). Ultimately, in the body, different EV categories are released from millions of individual cells, forming an immensely heterogeneous EV pool. This also results in the loss of information on their biogenesis and therefore, in practice, EVs are distinguished from one another by their size; small (sEV, <200 nm) or large (lEV, >200 nm), as described in the minimal information for studies of extracellular vesicles 2018 (MISEV2018) guidelines (7). Note that since there is a certain level of size overlap between the different subtypes, none of these subpopulations is exclusive for either the small or the large EV group (8).

Due to their biogenesis, EVs have some important biophysical properties. First, EVs are delineated by a phospholipid bilayer, effectively shielding their cargo (consisting of proteins, oligonucleotides (RNA and DNA), lipids, and metabolites) from the hostile EC environment, thereby, maintaining the functionality and stability of biomolecules (9). Therefore, the cargo can be considered to represent the molecular state of the parental cell at the moment of EV formation. In addition, (trans)membrane proteins are incorporated into the lipid bilayer, which, in turn, allows targeted signal transduction with distant EV receiving cells and/or allows the selective delivery of their cargo in such cells (10, 11). In physiological conditions, this cargo takes part in cellular communication and homeostasis (12, 13). In disease, however, the EV cargo can contribute to the pathology, for example by initiating a premetastatic niche at a distant location during cancer progression (14, 15).

Both of these EV characteristics are appealing for clinical implementations, offering great potential for both therapeutic applications (*i.e.* targeted drug delivery) as well as for biomarker discovery (*e.g.* disease diagnosis, prognosis, and therapy guidance) (16, 17, 18). Although the therapeutic applications remain rather hypothetical or, at best, experimental to date (19, 20), the biomarker potential of circulating EVs has known a substantial increase in the past decade. Indeed, EVs are present in different biofluids, such as plasma and urine, which can be isolated in a low to noninvasive way at multiple time points.

In general, considering the biophysical properties of EVs, the identification and characterization of protein biomarkers within or on circulating EVs from liquid biopsies might lead to (early) disease diagnosis, monitoring of disease progression and therapeutic response, all critical aspects toward the establishment of precision treatment (21). In this respect, EVs have shown clinical potential as a source of circulating biomarkers for different cancers (*e.g.* breast (18), urinary tract (22) and bone cancer (23)) and other diseases such as Alzheimer’s disease (24, 25), chronic liver disease (26) and multiple sclerosis (27).

Unfortunately, multiple challenges complicate EV protein research, especially with regard to proteome analysis by bottom-up mass spectrometry (MS), which is the method-of-choice for unbiased protein biomarker discovery (28). Indeed, once EVs are released by the cell they form a heterogeneous pool that resides in biological complex matrices that contain proteins at levels that exceed those of the low abundant EV proteins by several orders of magnitude (29), such as albumin in plasma and uromodulin in urine. In addition, they coexist with non-EV particles that overlap in size and density such as apolipoprotein particles. As a result, an adequate EV enrichment and purification strategy remains essential for in-depth EV-associated biomarker discovery (30). Of note, in MS-based proteomics, the recent advent of data-independent acquisition (DIA) MS due to computational (31, 32) and instrumental (33) improvements has proven to cope more successfully with the abovementioned challenges compared to traditional data-dependent acquisition, as it is less biased to relative high protein levels.

Over the years, different EV enrichment strategies have been developed to enrich, purify, and concentrate EVs for in-depth proteome analysis and quantitative profiling, with the most prominent strategies being based on size (ultracentrifugation (UC) and size-exclusion chromatography (SEC)) (34, 35), density (density-gradient UC) (36), precipitation (37, 38, 39), EV membrane interaction (EVTrap) (9, 40), or capture of molecular markers present on the EV surface (immunocapture) (41). To further enhance the purity of EV preparations, different orthogonal approaches can be combined (42, 43). However, a golden standard is yet to be determined, as the different strategies not only differ in the resource and time requirements, but also in terms of effectiveness and reproducibility. Indeed, the different EV isolation techniques introduce variation between experiments in terms of EV count and heterogeneity of molecular (protein) cargo. This has been emphasized in different studies in which EV enrichment strategies were compared (44, 45, 46, 47, 48). Along this line, initiatives such as MISEV and EV-TRACK were established within the EV research community to encourage transparency on experimental procedures used to enrich EVs and on acquired results, thereby enhancing interlab reproducibility (7, 49). Importantly, purified, recombinant EVs are now used as biological reference material to monitor technique development and reproducibility, and improve data normalization (50, 51). However, all enrichment strategies have one major bottleneck in common; they lack the possibility for parallelization and high-throughput set-ups. Indeed, enrichment strategies such as (density gradient) UC require (multiple) lengthy centrifugation steps and are generally limited to six or eight samples and require expensive instrumentation. SEC often requires a concentration step both prior and after EV enrichment and, in addition, also validation (often by Western blotting) to identify those fractions containing EV material (52). Commercial EV enrichment kits are again expensive and, more importantly, show high variability between suppliers (53, 54).

Ultrafiltration (UF) has found its place among other enrichment methods such as commercially available ExoDiscs (55) or in a flow-field set-up, especially for the isolation of small EVs (56, 57). However, as mentioned above, these approaches are sequential and therefore limited in throughput. In addition, it requires more specific instrumentation as opposed to centrifugation-driven UF (also referred to as dead-end or normal UF). UF has thus far been mainly used as an intermediate step in EV enrichment strategies to reduce the volume of conditioned medium (CM) or liquid biopsy either prior (*e.g.* density gradient UC) or after EV enrichment (*e.g*. SEC) (58, 59, 60). The main challenges associated with UF for EV proteome analysis are the inefficient removal of non-EV proteins, irreversible blocking of the filter membrane and low recovery of the retained particles (61, 62). However, it also offers inherent advantages, such as the limited cost, reduced sample preparation time, and the potential of analyzing multiple samples simultaneously using standard lab equipment. In addition, UF has little bias toward EV subpopulations (63), in contrast to (for instance) immunocapture.

The overall aim of our study was to develop an EV enrichment strategy that has the potential for fast and affordable high-throughput analysis, preferably using only standard instrumentation, yet is robust, specific and easy to use. Here, we propose an UF strategy for the isolation and purification of EVs from CM and bovine serum, which we termed filter-aided EV enrichment or filter-aided extracellular vesicle enrichment (FAEVEr). In short, precleared CM or serum is loaded on 300 kDa molecular weight cutoff (MWCO) filters and centrifuged briefly at a moderate speed during which EVs are retained on the filter, whereas the bulk of globular (non-EV) proteins do not. Remaining filter-retained non-EV proteins are removed through subsequent purification steps that include Tween-20-containing wash buffers. The retained (purified) EVs are then efficiently lysed on the filter in SDS-containing buffer and the EV lysates are conveniently collected by centrifugation and processed for proteomics applications, such as Western blotting and liquid chromatography coupled tandem mass spectrometry (LC-MS/MS) analysis. Recombinant EV (rEV) material isolated from HEK293T CM was used for testing and optimizing our strategy for comparison to UC, a widely implemented EV enrichment strategy. We demonstrate that the EV purity is considerably improved when including Tween-20 in the wash steps without jeopardizing EV integrity, even at high concentrations of the detergent. We then successfully applied FAEVEr to complex biofluids (bovine serum, human plasma, and human urine) and investigated the effect on the EV protein cargo when using serum-depleted medium on MCF7 cell cultures.

## Experimental Procedures

### Experimental Design and Statistical Rationale

Our study focusses on the isolation of EVs from complex medium for proteomics using 300 kDa MWCO filters, including Tween-20 in the wash buffer to purify the vesicles by removing high abundant interfering proteins. Optimization and proof-of-concept experiments were performed with recombinant EV particles, derived from Gag-eGFP transfected HE293T cells. Intact EV particles are retained and immediately lysed on the filter to collect the proteome. We compared the results with UC (two-sample *t* test, triplicates) followed by the analysis of the effect of five different percentages Tween-20 on the proteome, both for FAEVEr as for UC (multiple-sample testing, duplicates). Our application study was performed on CM of MCF7 cells grown in different percentages of serum (multiple-sample testing, duplicates). The results of the different statistical analyses were further analyzed using gene ontology (GO) and pathway analysis with a false discovery rate (FDR) cutoff (BH) of 0.05.

### Cell Culture and Preclearing the CM

All cell culture experiments were performed using standard protocol and lab equipment.

#### rEV Production

A complete protocol for the transformation and production of (rEV material is described in (51, 64). In short, approximately 4 million HEK293T cells of low passage number (<10) were seeded in a T75 falcon with Dulbecco's modified Eagle’s medium (DMEM, Gibco) containing 10% standard fetal bovine serum (FBS) and incubated for 48 h at 37 °C in 5% CO_2_. The HEK293T cells were transfected by adding a mixture of polyethyleneimine and the DNA bait construct (12×, pMET7- GAG-eGFP, Addgene #80605) in DMEM + 2% FBS for 6 h at 37 °C in 5% CO_2_. The medium was then discarded and replaced with 8 ml fresh DMEM supplemented with 10% EV-depleted FBS (EDS, Thermo Fisher Scientific, A2720801). After 48 h of incubation at 37 °C in 5% CO_2_, cell transfection was evaluated under UV light (excitation at 488 nm and emission at 507 nm) before the CM was isolated. The CM was immediately precleared by centrifugation at 1000*g* for 10 min and filtration of the supernatant using 0.22 μm filters (Millex). The precleared CM was divided over different aliquots and frozen in −80 °C until further use. Note that pure rEV extracts are commercially available (SAE0193, Sigma-Aldrich).

#### MCF7 Cells

MCF7 cells of low passage number (<10) were cultured using a standard protocol and equipment. The cells were seeded in T75 flasks with DMEM containing 10% standard FBS and incubated at 37 °C in 5% CO_2_. When the cells reached 70 to 80% confluence, the medium was discarded and the cells were washed three times with phosphate buffered saline (PBS). Experiments were performed in duplicate, adding 8 ml of DMEM supplemented with either 0%, 2%, 5%, or 10% EDS (Thermo Fisher Scientific, A2720801). After 24 h, the CM containing 0% EDS were isolated, whereas the 2%, 5%, and 10% EDS containing CM were isolated after 48 h. The CM was immediately precleared by two centrifugation rounds (500*g* for 5 min, and 10,000*g* for 10 min, room temperature) and filtration using 0.22 μm filters (Millex).

### Plasma and Urine Collection

Plasma and urine were collected from the same male control subject (Caucasian, 34 years) after signing an informed consent form approved by the ethical commission of University hospital Ghent (EC-2019_1506), in accordance with the Declaration of Helsinki. Subsequently, 10 ml blood was collected in EDTA tubes and centrifuged at 2000*g* for 10 min (room temperature), after which the supernatant was collected and the centrifugation repeated. The collected plasma was stored in EDTA tubes per 1 ml aliquots at −80 °C. Minutes before EV isolation, 3 ml plasma was thawed in a water bath (room temperature), centrifuged at 10,000*g* for 10 min and the supernatant was filtered using 0.22 μm filters (Millex). A urine sample was collected midstream in the late afternoon in a standard 50 ml falcon and immediately centrifuged for 10 min at 10,000*g* (room temperature) followed by filtration using 0.22 μm filters (Millex) and processed within the same day.

### EV Enrichment

#### Ultrafiltration Using 300 kDa MWCO Filters

In addition, 1 ml or 3 ml of precleared CM (for rEVs and MCF7 cell conditioned medium, respectively) was diluted 1:1 with PBS with or without supplemented Tween-20 and vortexed for 5 to 10 s. For human plasma, 1 ml or 500 μl was diluted 1:5 or 1:10 with PBS, respectively, in technical duplicates. Human urine sample remained undiluted and was split over technical duplicates, 20 ml each. The final volume of the CM and plasma was then transferred to 300 kDa MWCO polyethersulfone (PES) Vivaspin6 filters (Sartorius, VS0652) and the urine was transferred to 300 kDa MWCO PES Vivaspin20 filters (Sartorius, VS2051) and centrifuged. Centrifugation was done for 10 to 15 min at room temperature either at 6000*g* for fixed angle rotors (F14 x 50cy, Sorvall Lynx 4,000, Thermo Fisher Scientific) or at 4000*g* for swing-out rotors (*e.g.* A-4-44, 5804R, Eppendorf). The filter retentate was washed three times with 1 ml wash buffer (PBS with or without supplemented Tween-20) and centrifuged for 5 to 7 min per wash step. After three rounds of washing, the retained particles were either solubilized from the filter for nanoparticle tracking analysis (NTA) or electron microscopy, or immediately lysed on the filter for maximal recovery of the proteome. For recovery, 500 μl 50 mM Hepes was added on the filter and vortexed for 10 to 15 s after which the solution was transferred to a fresh tube and vacuum-concentrated (SpeedVac). To immediately collect the EV lysate, 5% SDS in 50 mM triethylamine bicarbonate (TEAB) is added and collected after 15 min incubation (37 °C, 500 rpm) by centrifugation (5 min at room temperature).

#### Ultracentrifugation

Subsequently, 1 ml of precleared CM (for rEVs) was transferred to polycarbonate thick-wall tubes (Beckman Coulter, 343778) and centrifuged for 90 min at 100,000*g* (4 °C) using a Optima TLX ultracentrifuge equipped with a TLA-120.2 rotor (Beckman Coulter). The supernatant was carefully removed and the pellet washed with PBS with or without Tween-20 before a second round of UC for 90 min at 100,000*g* (4 °C). Again, the supernatant was carefully removed, and the pellets were either solubilized for NTA or electron microscopy, or immediately lysed for maximal recovery of the proteome. The EVs were lysed by adding 5% SDS in 50 mM TEAB, and the lysate was collected after 15 min incubation (37 °C, 500 rpm).

### Protein Analysis

#### Western Blot

Samples for Western blot analysis were incubated for 5 min at 95 °C with XT sample buffer (4×) and XT reducing agent (20×) (Bio-Rad). The protein material was separated by SDS-PAGE (4%-12%) for 80 min at 120 V prior to transfer onto a polyvinylidene fluoride membrane (100 V, 30 min). The membrane was incubated overnight at 4 °C with primary antibodies (rabbit monoclonal anti-Calnexin (ab133615) and mouse monoclonal anti-HIV p24 39/5 4A (ab9071); 1:1000). The next day, the membrane was washed several times before incubation for 1 h at room temperature with secondary antibodies (goat anti-rabbit IRDye 680 CW and goat anti-mouse IRDye 800 CW; 1:10,000). Visualization was done on an Odyssey infrared imager (v3.0.16, LI-COR Biosciences).

#### LC-MS/MS Sample Preparation

The proteome was prepared for LC-MS/MS analysis using S-Trap mini columns (ProtiFi, C02-mini-80) using the manufacturer’s protocol. In brief, proteins in lysis buffer (5% SDS, 50 mM TEAB, pH 7.4) were reduced and alkylated using 5 mM tris(2-carboxyethyl)phosphine (10 min at 55 °C) and 20 mM iodoacetamide (10 min at room temperature in the dark), respectively. The sample was acidified to 1.2% phosphoric acid and diluted seven-fold with binding/wash buffer (90% methanol in 100 mM TEAB, pH 7.4). The samples were transferred to the corresponding S-Trap columns and each time centrifuged at 4000*g* for 30 s during sample loading and washing. For protein digestion, 0.5 μg of trypsin (Promega, V5111) was added to 125 μl 50 mM TEAB (pH 7.4), and samples were incubated overnight at 37 °C. The next day, the peptides were recovered by sequential elution by centrifugation (4000*g* for 30 s at room temperature) using 80 μl 50 mM TEAB (pH 7.4), 80 μl 0.2% formic acid, and 80 μl of 50% acetonitrile (ACN), 0.2% formic acid in dH_2_O. The eluates (app. 365 μl) were transferred to MS vials, vacuum-dried in a SpeedVac and stored at −20 °C. We have submitted all relevant data of our experiments to the EV-TRACK knowledgebase (EV-TRACK ID: EV240045) (34).

#### LC-MS/MS Analysis

Purified peptides were redissolved in loading solvent A (0.1% TFA in water/ACN (98:2, v/v)) and the peptide concentration was determined on a Lunatic instrument (Unchained Labs). For each sample the injection volume was adjusted to inject equal amounts of peptide material (500 ng) for LC-MS/MS analysis on an Ultimate 3000 RSLCnano system in-line connected to a QExactive Exploris (for optimization and validation) or an Orbitrap Fusion Lumos mass spectrometer (Thermo Fisher Scientific) (MCF7 cells and EVs). QCloud was used to control instrument longitudinal performance during the project (57, 58). The MS proteomics data have been deposited to the ProteomeXchange Consortium via the PRIDE (59) partner repository with the dataset identifiers PXD051938 (comparison FAEVEr with UC), PXD051955 (comparison MCF7 proteome of EVs and cells under starving conditions), PXD051956 (comparison FBS with EDS) and PXD056479 (exploratory study of human plasma and urine). In addition, all spectra were uploaded to Panorama (Skyline).

### LC-MS/MS Analysis using the Fusion Lumos Instrument

LC-MS/MS DIA on the Fusion Lumos was initiated by trapping the peptide material at 20 μl/min for 2 min in loading solvent A on a Pepmap column (300 μm internal diameter (I.D.), 5 μm beads, C18, Thermo Fisher Scientific). The peptides were separated on a 110 cm μPAC prototype column (Thermo Fisher Scientific), kept at a constant temperature of 50 °C. Peptides were eluted by a non-linear gradient starting from 2% MS solvent B (0.1% formic acid (FA) in acetonitrile), reaching 26.4% MS solvent B in 82 min and 44% MS solvent B in 90 min and 56% MS solvent B in 100 min starting at a flowrate of 600 nl/min for 5 min, and completing the run at a flow rate of 300 nl/min, followed by a 5-min wash at 56% MS solvent B and re-equilibration with MS solvent A (0.1% FA in water). The mass spectrometer was operated in data-independent mode. Full-scan MS spectra ranging from 400 to 900 m/z without overlap. A target value of 4E5 was set with a maximum fill time of 50 ms and a resolution of 60,000, followed by 50 quadrupole isolations with a precursor isolation width of 10 m/z for higher energy collisional dissociation fragmentation at an NCE of 34% after filling the trap at a target value of 4E5 for 54 ms (maximum injection time). MS2 spectra were acquired at a resolution of 30,000 at 200 m/z in the Orbitrap analyzer with a scan range of 200-1800 m/z in the Orbitrap analyzer without multiplexing.

### LC-MS/MS Analysis Using the Orbitrap Exploris 240 Instrument

The peptide material was injected for LC-MS/MS analysis on a Vanquish Neo UHPLC System in-line connected to an Orbitrap Exploris 240 mass spectrometer (Thermo Fisher Scientific). Injection was performed in trap-and-Elute workflow in combined Control mode (maximum flow of 60 μl/min and maximum pressure of 800 bar) in weak wash solvent (, 0.1% trifluoroacetic acid in water/ACN (99.5:0.5, v/v)) on a 5 mm trapping column (Thermo Fisher Scientific, 300 μm internal diameter (I.D.), 5 μm beads). The peptides were separated on a 250 mm Aurora Ultimate, 1.7 μm C18, 75 μm inner diameter (IonOpticks) kept at a constant temperature of 45 °C. Peptides were eluted by a gradient starting at 0.5% MS strong wash solvent (SW) (0.1% FA in acetonitrile) reaching 26% MS SW in 30 min, 44% MS SW in 38 min, and 56% MS SW in 40 min followed by 5-min wash at 56% MS SW and column equilibration in pressure control mode (separation column: fast equilibration, maximum pressure of 1500 bar, equilibration factor=2; Trap column: fast wash and equilibration, wash factor=100) with MS weak wash. The flow rate was set to 300 nl/min. The mass spectrometer was operated in data-independent mode, automatically switching between MS and MS/MS acquisition. Full-scan MS spectra ranging from 400 to 900 m/z with a normalized target value of 300%, a maximum fill time of 25 ms and a resolution at of 60,000 were followed by 30 quadrupole isolations with a precursor isolation width of 10 m/z for higher energy collisional dissociation fragmentation at an NCE of 30% after filling the trap at a normalized target value of 2000% for maximum injection time of 45 ms. MS2 spectra were acquired at a resolution of 15,000 with a scan range of 200 to 1800 m/z in the Orbitrap analyzer without multiplexing. The isolation intervals were set from 400 to 900 m/z with a width of 10 m/z using window placement optimization. EASY-IC was used in the start of the run as internal mass calibration.

#### Database Searching and Data Analysis

DIA spectra were searched with the DIA-NN software (https://doi.org/10.1038/s41592-019-0638-x; v1.8.2 b) (31) in library-free mode against the combination of two protein databases downloaded from Swiss-Prot; the complete human protein sequence database (January 2021, 20,394 sequences) supplemented with bovine serum protein sequences (March 2021, 618 sequences). For searches that included rEV material, we included the Gag-eGFP protein sequence as well. The mass accuracy was set to 10 ppm and 20 ppm for MS1 and MS2, respectively, with a precursor FDR of 0.01. Enzyme specificity was set to trypsin/P with a maximum of two missed cleavages. Variable modifications were set to oxidation of methionine residues (to sulfoxides) and acetylation of protein N termini. Carbamidomethylation of cysteines was set as a fixed modification. The peptide length range was set to 7 to 30 residues with a precursor charge state between 1 and 4 and an m/z range between 400 to 900 and 200-1800 for the precursor and fragment ions, respectively. Cross-run normalization was set to retention time dependent with the quantification strategy set to high accuracy and the neural network classifier to single-pass mode. The result file was further processed in KNIME (https://www.knime.com/; v4.3.3) by removing nonproteotypic peptides and identifications with a protein q-value above 0.01. Peptide quantifications were aggregated to protein group quantifications using the median of the corresponding normalized label-free quantification values. Further data analysis was performed with Perseus (version 1.6.14.0) (65), GraphPad Prism (https://www.graphpad.com/; v9.4.1), and RStudio (https://rstudio-desktop.en.download.it/; 2023.12.1). iBAQ values were generated with the DIAgui shiny app (66) using the default settings.

For the analysis of the EV and cellular proteomes across different experimental conditions, proteins that were significantly differentially abundant were identified by multiple sample testing (ANOVA) and selected for hierarchical clustering (Euclidean distance with averaged linkage) after Z-scoring the individual proteins over the different conditions. Row clusters were defined manually and used for GO enrichment against the corresponding initial database and included biological process, molecular function, cellular component annotations, and Kyoto Encyclopedia of Genes and Genomes pathways. GO term enrichment was done using the online WebGestalt tool for gene ontology enrichment analysis (67) and string database (StringDB.org).

### EV Characterization

#### NTA Analysis

Recuperated EV particles were diluted to 1 ml using PBS (pH 7), injected in a calibrated Zetaview and analyzed with the corresponding software package (version 8.05.16 SP2). The temperature was maintained at 23 °C, and the analysis was done at a sensitivity of 70 and a shutter of 100 for three cycles at 60 fpm. The results were plotted and analyzed in GraphPad Prism (https://www.graphpad.com/updates/prism-10-2-2-release-notes; v10.2.2).

#### Scanning Electron Microscopy

Filters containing EVs were placed in 2 ml Eppendorf tubes and fixed with 4% paraformaldehyde and 2% glutaraldehyde in 0.1 M sodium cacodylate buffer at room temperature. After 1 hour, the fixative was replaced and filters were washed three times for 5 min with dH_2_O. After dehydration in 30%, 50%, 70%, 95%, and 2× 100% ethanol for 30 min each, the filters were critical point dried (EM CPD300, Leica) and imaged on a Crossbeam 540 scanning electron microscopy (SEM) (Zeiss) at 1 kV.

#### Transmission Electron Microscopy

Aliquots (5 μl) of the EV solutions were blotted for 1 min on formvar- and carbon-coated Ni maze grids (EMS), which were glow discharged for 40 s at 15 mA. The grids were then washed five times in droplets of dH_2_O and stained in a droplet of 1/4 Uranyl Acetate Replacement Stain (EMS)/dH_2_0 for 1 min. Excess stain was removed with filter paper and the grids were air dried for at least 4 h before viewing with the transmission electron microscopy (TEM). Imaging was done at 80 kV on a JEM1400plus (JEOL).

## Results

### Use of 300 kDa MWCO Filters to Enrich EVs

Although UF is a valid and well-implemented method to concentrate intact EVs, it holds many challenges as a stand-alone EV enrichment strategy. Nevertheless, UF also has important advantages such as low cost and fast processing times, and it does not require specific instrumentation compared to, for instance, UC or tangential flow filtration.

**Fig. 1:**
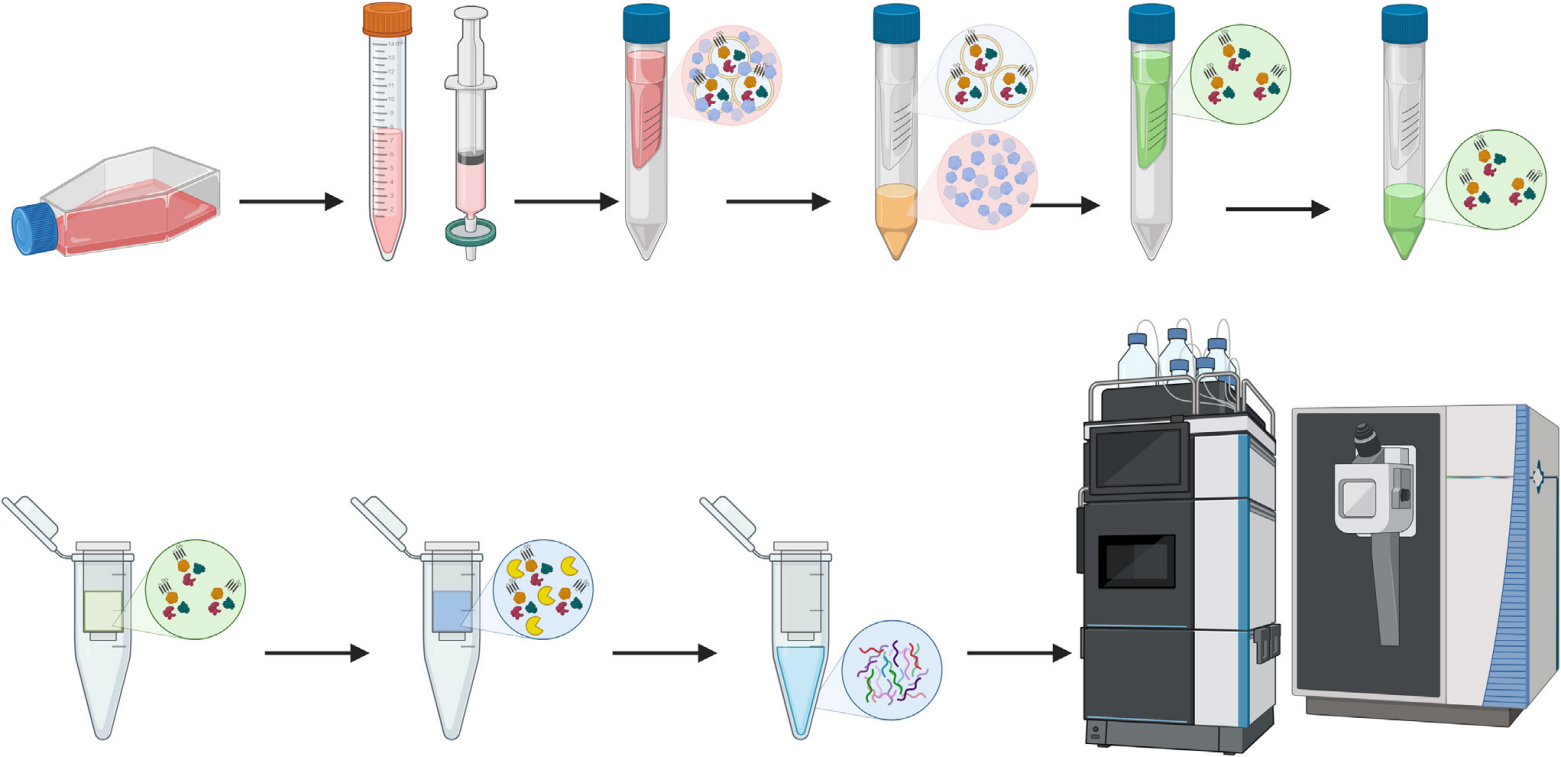
**Schematic overview of the FAEVEr workflow**. Conditioned media or biofluids are precleared and transferred onto the 300 kDa MWCO PES filter (*red*). Driven by centrifugation, the globular proteins pass through the filter (*pink*), whereas the EV particles are quantitatively retained (*orange*). The retentate is subsequently washed three times using PBS supplemented with Tween-20 (*blue*) to further increase the EV purity. Ultimately, intact particles are recovered for characterization, or lysed (*green*) immediately on the filter. The lysate is recovered by centrifugation and prepared for proteomic applications (*e.g.* LC-MS/MS analysis or Western blotting). Figure created in Biorender. EV, extracellular vesicle; FAEVEr, filter-aided extracellular vesicle enrichment; LC-MS/MS, liquid chromatography coupled tandem mass spectrometry; MWCO, molecular weight cutoff; PES, polyethersulfone.

Here, we introduce FAEVEr, a strategy that uses 300 kDa MWCO PES filters to purify and concentrate intact EVs (Fig. 1). In brief, precleared conditioned media or biofluids are transferred onto the 300 kDa MWCO filter membrane and centrifuged at moderate speed (4000–6000*g*), retaining the EV particles on the filter (retentate) whereas globular non-EV proteins are removed (filtrate). By including three consecutive wash steps, non-EV proteins are increasingly removed, by which the EV retentate is further purified. Eventually, by using a lysis buffer with high percentages (5%) of SDS, EVs undergo efficient lysis, and their cargo is conveniently recovered by centrifugation. The EV sample is then further processed for proteomics using S-Trap columns (68). We opted for 300 kDa MWCO PES membranes for several reasons. First, a pore size of 300 kDa MWCO allows a more confluent flow-through of (bio)fluids at a lower centrifugation speed compared to smaller pore sizes, including 100 kDa MWCO filters often used for concentration steps in SEC and dgUC. In addition, 300 kDa MWCO filters have an average pore size of 30 to 35 nm (according to the manufacturer), which is well below the average sEV size (120 nm), yet above 99% of the size (MW) of contaminating globular proteins (and even protein complexes) and was reported to isolate EVs efficiently (69). Secondly, as we lyse the purified EVs on the filter in buffers with high percentages of SDS, we recover virtually all possibly retained protein material from the filter membrane by centrifugation. Therefore, we expect (near-) complete recovery of the EV proteome from the filter. Thirdly, PES membranes are hydrophilic with low-protein adsorption characteristics, resistant against high pH ranges and temperatures, and can withstand a wide variety of solvents, buffers, and detergents. Finally, to our knowledge, no 300 kDa MWCO filters are available in materials other than PES, including regenerated cellulose.

rEV material was used for the initial optimization of the 300 kDa MWCO UF strategy as it has similar biochemical and biophysical characteristics as sample EVs (50). The rEV material is derived from the CM of HEK293T cells, cultured in 10% EV-depleted serum, that overexpress the Gag-eGFP fusion protein (51). The Gag subunit multimerizes at the plasma or endosomal membrane and initiates membrane curving, resulting in the formation of microvesicles and intraluminal vesicles, respectively, commonly referred to as virus-like particles (64, 70) (Supplemental Fig. S1). As a result, secreted rEV material is overrepresented in the CM and contains high levels of luminal Gag-eGFP, enabling the evaluation of EV integrity during the consecutive FAEVEr steps.

**Fig. 2:**
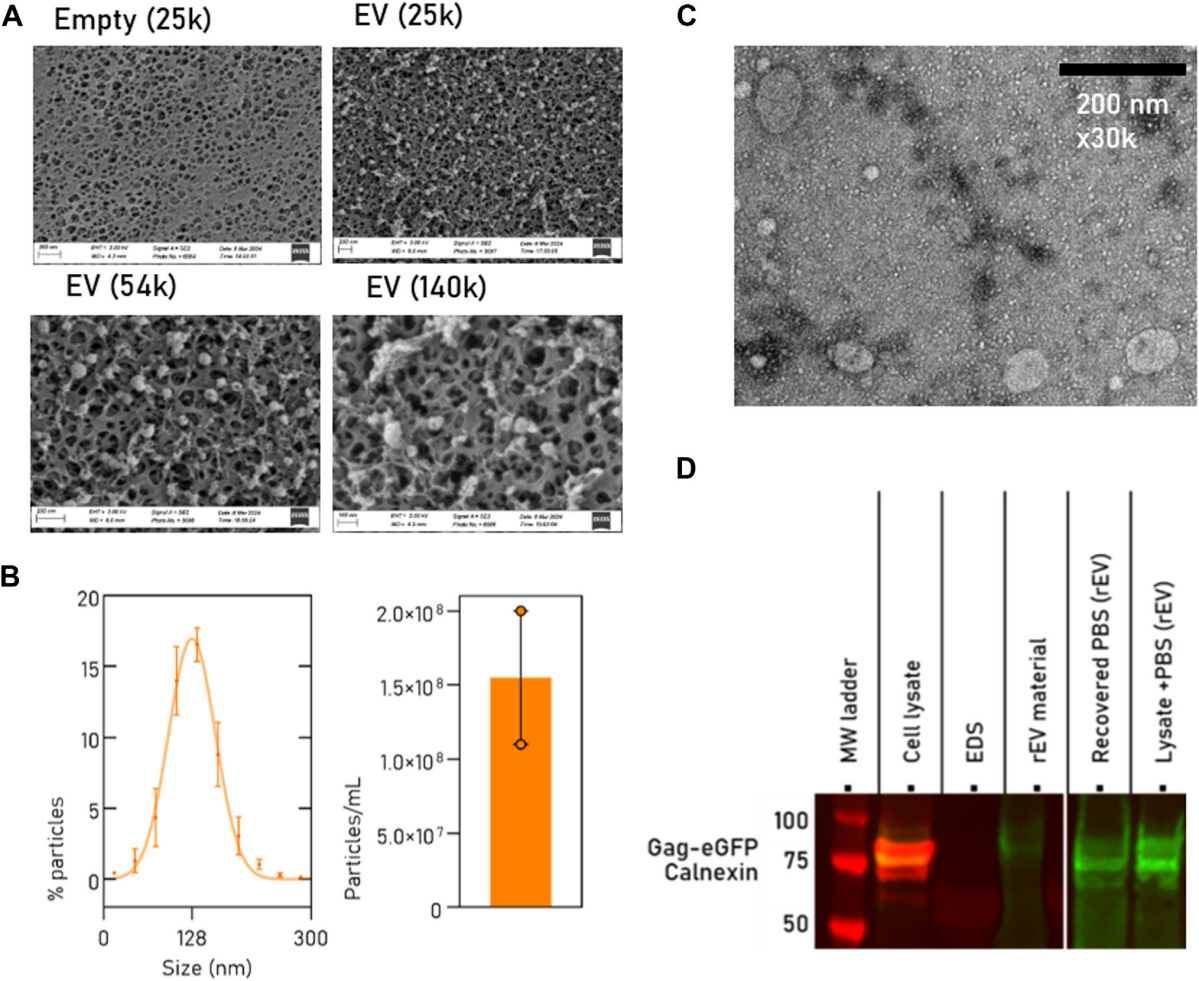
**Qualitative analysis of FAEVEr retained particles**. *A*, scanning electron microscope of an empty filter and after rEV enrichment. *B*, EV size distribution and count, and transmission electron microscopy (*C*) of recovered rEV particles from the filter. *D*, Western blot reveals incomplete recuperation of rEV particles from the filter. EDS, EV-depleted serum (negative control), rEV material, commercial rEV particles (positive control), PBS, phosphate-buffered saline washed rEV particles. EV, extracellular vesicle; FAEVEr, filter-aided extracellular vesicle enrichment; rEV, recombinant EV.

We confirmed that rEV particles are retained on the 300 kDa MWCO mesh using SEM (Fig. 2*A* and Supplemental Fig. S2), indicating that UF is an efficient EV enrichment strategy, resembling the results of commercial ExoDisc (55). Indeed, after completing the FAEVEr protocol, rEV particles were recovered from the filter for NTA (Fig. 2*B*) and TEM characterization (Fig. 2*C*). However, in line with Vergauwen *et al.* (61), we noticed that the quantitative recuperation of particles from the filter PES membrane is variable and that not all particles are recovered. When the remaining rEV fraction on the filter was lysed and analyzed on Western blot, we observed that a substantial share of the rEV material fails to be solubilized and remains on the filter (Fig. 2*D*).

After lysis and LC-MS/MS sample preparation using S-Trap columns (68, 71), the FAEVEr-derived EV proteome was compared to UC, which is the most widely applied strategy for EV enrichment according to EV-TRACK (49). Both strategies resulted in a high number of total identifications, with a 15% increase for FAEVEr (Supplemental Fig. S3, *A*–*C*), and a high (85%) overlap between the two strategies (Supplemental Fig. S3*D*). Of importance, we observed a steep decrease of the accompanying coefficient of variance (%CV) for FAEVEr between the replicates as well as fewer missing values, which results in an increased data completeness and therefore higher reproducibility with more accurate protein quantifications (Supplemental Fig. S3, *E* and *F*). As the CM contained 10% EV depleted FBS (EDS), bovine precursor intensities were used as a direct measure of contamination by comparing the occupancy of their peptide ions in the LC-MS/MS spectral space to that of human derived precursors. We found that bovine precursor abundance in both strategies remained high (30% and 35%, respectively).

Concluding this part, we established that FAEVEr is an efficient approach for isolating intact EV particles (Fig. 2, *A*–*C*) from CM for proteome analysis. Although we can only recuperate part of the EV particles from the filter (Fig. 2*D*) for characterization purposes, for EV proteomics, we circumvented this poor recuperation by directly lysing the retained EV particles on the filter membrane using high percentages of SDS. This ensures complete EV lysis, comprehensive solubilization of (membrane) proteins (72) and maximal protein recovery from the filter membrane. Note that SDS is irreconcilable with LC-MS/MS analysis and we therefore introduced S-Trap columns in our workflow. The S-Trap protocol is easy, fast and reproducible and has been extensively compared to other methods in different studies (73, 74). Although the results are promising and competitive with UC, the purity of the EV proteome remained unsatisfactory.

### Tween-20 Improves the Removal of Non-EV Proteins

One major pitfall of UF is the inefficient removal of non-EV proteins, which has a detrimental effect on the numbers of identified and quantified EV proteins following LC-MS/MS analysis. Therefore, we explored the use of mild detergents to minimize the interaction between proteins and the filter membrane. However, as EVs are delineated with a lipid bilayer and therefore susceptible to detergent-mediated lysis, caution should be taken with the choice and concentration of the detergent. A study by Osteikoetxea *et al.* explored the minimal concentration of different detergents required for efficient lysis of EV particles in solution (75) and found that this concentration greatly depends not only on the detergent used, but also on the EV subpopulation (exosomes, microvesicles, and apoptotic bodies). In particular, the smallest population (exosomes) demonstrated increased resilience against detergents compared to larger vesicles (microvesicles and apoptotic bodies) due to an increase in liquid order because of an accumulation of cholesterol and sphingolipids during their formation (76, 77, 78). In their work, Osteikoetxa *et al.* demonstrate that EVs are generally efficiently lysed even at low percentages of SDS, Triton X-100 or sodium deoxycholate. On the contrary, an exceptional high tolerance is observed towards Tween-20, a rather mild detergent that is commonly used in other biochemical assays such as Western blotting to reduce unwanted adsorption of protein material to membranes (79) and thereby decreases irreversible or total membrane fouling (80, 81). Therefore, Tween-20 was explored here as an additive to improve the efficiency of the washing steps and thus removal of non-EV proteins during the enrichment of EVs while keeping EVs intact. The efficiency of washes with Tween-20-containing buffers was tested by Coomassie staining of the different filtrate fractions of CM after completing the FAEVEr protocol using 0%, 0.1%, 0.5%, 1%, 2%, and 5% Tween-20 in the wash buffer. We observed that PBS-only (the 0% Tween-20) condition is not capable of removing the bulk of bovine serum albumin ( 65 kDa), whereas bovine serum albumin-bands are more intense in the Tween-20 supplemented wash fractions. In addition, upon recovery of the retained material by lysis in 5% SDS, we observed an intense band for the PBS only condition, which was absent in the Tween-20 supplemented ones (Supplemental Fig. S4). This suggested that Tween-20 is indeed capable of removing the bulk of (contaminating) bovine serum material from the filter membrane and therefore should result in an EV proteome fraction that is more pure.

**Fig. 3:**
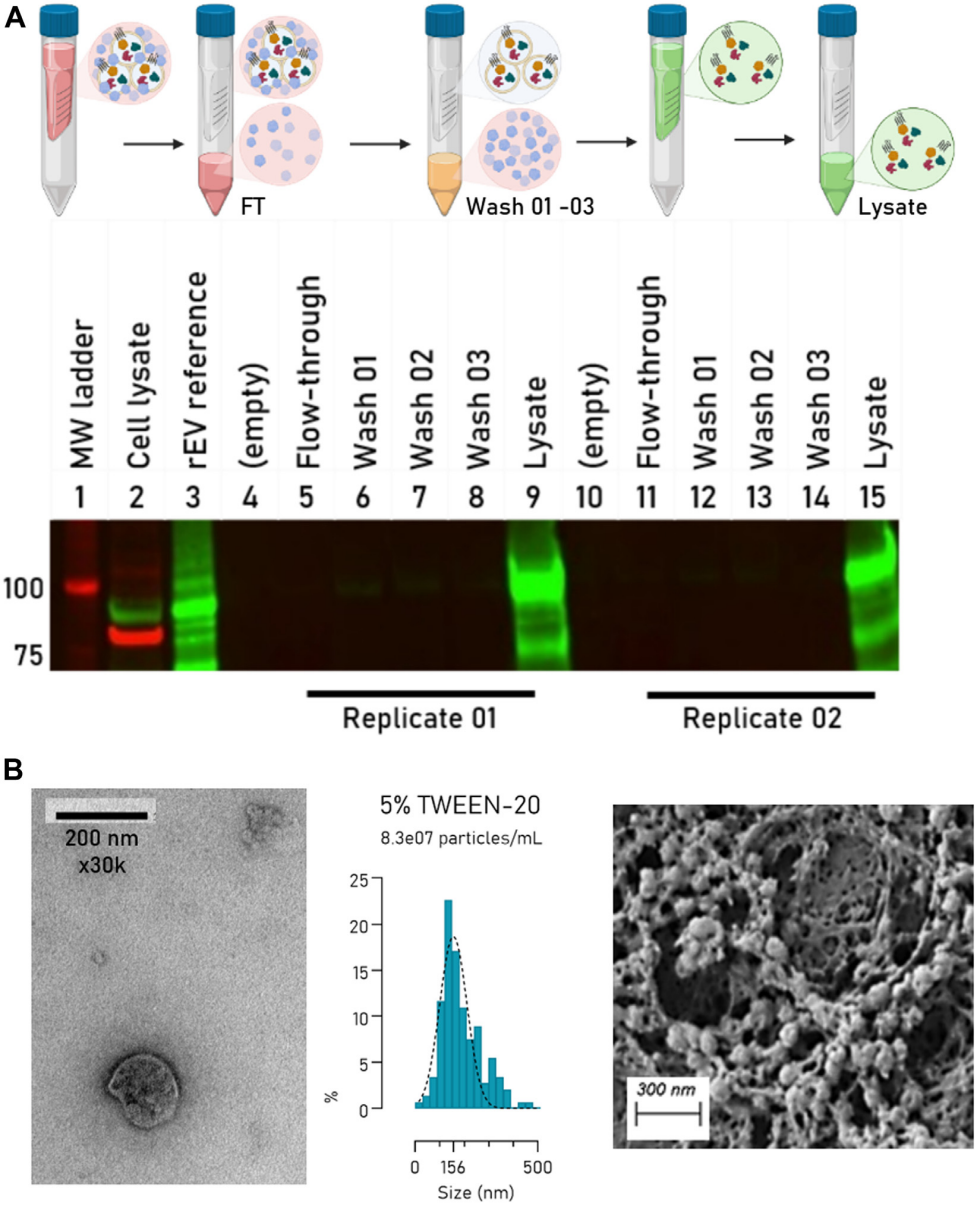
**The integrity of rEV particles remains even when washing with buffers supplemented with 5% Tween-20**. *A*, Western blot of the different fractions during FAEVEr showing that no (or just marginal) luminal Gag-eGFP is observed in the flow-through and wash steps, implying that EVs remain intact. In addition, a strong signal is observed in the lysate fractions, indicating that we can efficiently recover the rEV proteome. *B*, qualitative analysis by TEM, NTA, and SEM of recovered rEV particles washed in PBS supplemented with 5% Tween-20. EV, extracellular vesicle; FAEVEr, filter-aided extracellular vesicle enrichment; NTA, nanoparticle tracking analysis; rEV, recombinant EV; SEM, scanning electron microscopy; TEM, transmission electron microscopy.

Next, we validated this beneficial effect of Tween-20 in wash buffers using rEV material, which also allows us to validate the integrity of the particles as they contain the Gag-eGFP protein on the luminal side. We thus argued that, when the rEV particles remain intact, no luminal Gag-eGFP should be present in the filtrates. In short, precleared CM containing rEV material was filtered (300 kDa MWCO) and washed several times with up to 5% Tween-20 in PBS. After each centrifugation step, the filtrate was collected and the presence of luminal Gag-eGFP validated on Western blot, shown in Figure 3*A*. The almost complete absence of luminal Gag-eGFP in the flow-through and washes (1, 2, 3) does not only indicate that intact rEV particles are retained on the filter, but also that they maintain their integrity in concentrations up to 5% Tween-20, as validated by TEM, SEM, and NTA (Fig. 3*B*). In addition, it also indicates that the proteome of retained EVs is efficiently recovered from the filter for downstream proteomic applications using a lysis buffer containing 5% SDS.

**Fig. 4:**
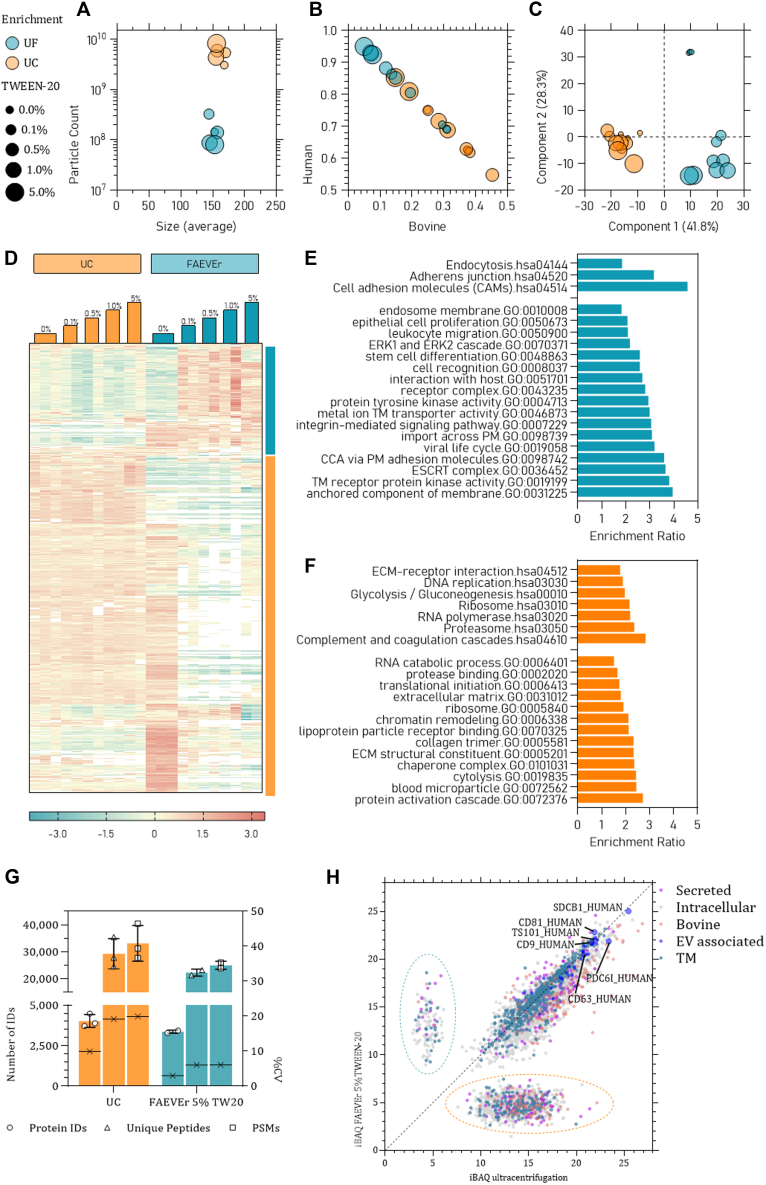
**Overview of the effect of Tween-20 at different percentages on EV enrichment by UC and FAEVEr.***A*, relative share of bovine and human precursor abundance as a measure of serum contamination in function of the enrichment strategy and the percentage of Tween-20 in the wash buffer. *B*, PCA plot of the shared protein intensities between the different experimental setups reveals three major groups. *C*, number and size of particles isolated by UC and FAEVEr. *D*, heatmap of the significantly differential abundant proteins (multiple sample test, *p*-value <0.05) per experimental setup with the accompanying GO analysis for FAEVEr enriched (*E*) and UC enriched (*F*) proteins. *G*, comparison of the number of identifications between conventional UC and FAEVEr with 5% Tween-20. *H*, comparison of the iBAQ intensities between conventional UC and FAEVEr with 5% Tween-20. Although the larger part of the proteins have a comparable intensity (including EV markers), a large population is uniquely quantified in UC (*orange circle*) that is associated with intracellular organelle lumen (including nuclear lumen, ER, and Golgi apparatus), intracellular transport, RNA and DNA processing, EC matrix constituent or were from bovine origin. CCA, cell-cell adhesion; EC, extracellular; ECM, extracellular matrix; ER, endoplasmic reticulum; EV, extracellular vesicle, FAEVEr, filter-aided EV enrichment (*teal*); GO, gene ontology; PCA, principal component analysis; TM, transmembrane; UC, ultracentrifugation; UC, ultracentrifugation (*orange*).

We further extended our comparison between FAEVEr and UC to evaluate the effect of different percentages Tween-20 on the purity of the final rEV proteome. NTA analysis of the enriched particles revealed that neither the enrichment strategy, neither the percentage of Tween-20 in the wash buffer had drastic effects on the particle size (around 150 nm), yet a noticeable difference in particle count was observed (Fig. 4*A*), probably due to the incomplete recovery from the filter membrane, as discussed earlier. We initiated the proteome analysis by comparing the spectral space distribution between human and bovine precursors. Here, we did not only observe a remarkable decrease in bovine precursor intensities using FAEVEr even at the lowest (0.1%) Tween-20 concentration, but also that increasing the Tween-20 concentration further improved this (Fig. 4*B*), a trend that is far less pronounced in UC. This resulted in three distinct groups following principle component analysis (Fig. 4*C*) which is also observed after conducting a multiple sample test; UC with or without Tween-20, FAEVEr without Tween-20 and FAEVEr with Tween-20 (Fig. 4*D*). Examination of the GO and Kyoto Encyclopedia of Genes and Genomes terms associated with the corresponding proteins revealed that in FAEVEr supplemented with Tween-20, terms including cell-cell adhesion, ESCRT mechanism and (trans)membrane proteins were more abundant (Fig. 4*E*). On the other hand, we noticed that in UC and FAEVEr without Tween-20, terms including RNA binding, cell cycle, proteasome, and metabolic activities were more prominent (Fig. 4*F*) as well as serum-related terms (*e.g.* blood microparticle and activation cascade). FAEVEr in combination with 5% Tween-20 appeared to be optimal yet showed a noticeable decrease in protein identifications compared to UC (Fig. 4*G*). We further assessed this by comparing the absolute protein abundance (iBAQ) and found that over 2700 proteins correlated well (R^2^ = 0.74), including six EV markers being equally abundant between the two strategies (Fig. 4*H*). Interestingly, bovine proteins and secreted human proteins were generally higher abundant in UC. Remarkably, over 1100 proteins were uniquely quantified in UC that were predominantly associated with the intracellular organelle lumen (including nuclear lumen, endoplasmic reticulum, and Golgi apparatus), intracellular transport, RNA and DNA processing, EC matrix constituent or were from bovine origin.

In conclusion, our results indicate that FAEVEr in combination with Tween-20 in the washing buffer improves the overall EV proteome purity, whereas this effect is less prominent in UC. Furthermore, we found that including 5% Tween-20 in the wash buffer resulted in optimal results.

### Effect of Serum Depletion (on the EV Proteome)

FBS is used as a standard supplement in cell culture media. Unfortunately, it is irreconcilable with EV analysis from conditioned cell media as it contains EVs from bovine origin that interfere with proteome analysis. Therefore, EDS alternatives are used. Consequently, researchers often expand cells for several days in media containing EDS or even refrain from adding serum. To this day, it remains a controversial topic (82) with some researchers advocating against the use of serum because it remains a potential source of xeno-contamination. Others deem serum as an essential supplement for normal cell growth with serum depletion stressing cells due to starvation (83, 84), which potentially biases the proteome of the cell culture-derived EVs. Benefiting from the EV-TRACE repository, we noticed that between 2018 and 2023 approximately 56% of the EV experiments that were conducted on CM of human cell lines were performed using EDS, whereas 30 to 35% of the EV experiments was performed using serum-free conditions.

Following the MISEV2018 (7) guidelines, we investigated whether the used EDS is indeed devoid of EV material and addressed this using FAEVEr comparing EDS with FBS. In addition, we examined uses of FBS or EDS had an effect on the cellular and the EV proteome. For these experiments, we made use of MCF7 breast cancer cells.

As the high protein concentrations and the accompanying viscosity of serum samples may result in low filtration speed and irreversible blocking of the filter membrane, we diluted the sample three times prior to filtration. After completing the FAEVEr protocol, the proteomes of the FAEVEr filter-retained fraction from commercial FBS and EDS were compared by LC-MS/MS. We validated that EV-specific molecular markers were absent in EDS as well as proteins associated with EV biogenesis. In contrast, these proteins were found abundantly in the FBS retained fraction (Supplemental Fig. S5).

In conclusion, using FAEVEr, we here provide evidence at the LC-MS/MS level that EDS is indeed devoid of EV material and that the FAEVEr strategy is capable of enriching EV particles from complex biological fluids such as (bovine) serum.

The protein concentration of serum is reduced by a factor six because of the EV depletion process (Supplemental Fig. S6*A*). Indeed, FBS is diluted several times to prevent unintended protein aggregation and pelleting by UC to remove EVs due to the high viscosity of undiluted FBS (74). We decided to explore the effect on the MCF7 cellular proteome once the medium was exchanged from 10% FBS to 10% EDS for 3 days. Besides no change in cell morphology by visual inspection or a significant change in cell count or cell death (Supplemental Fig. S6*B*), the LC-MS/MS analysis indicated that only 10 out of more than 7000 quantified proteins appeared to be significantly differentially regulated between the two conditions (Supplemental Fig. S6, *C* and *D*). We conclude that shifting from FBS to EDS has close to no influence on the viability, growth, or proteome of MCF7 cells in culture.

**Fig. 5:**
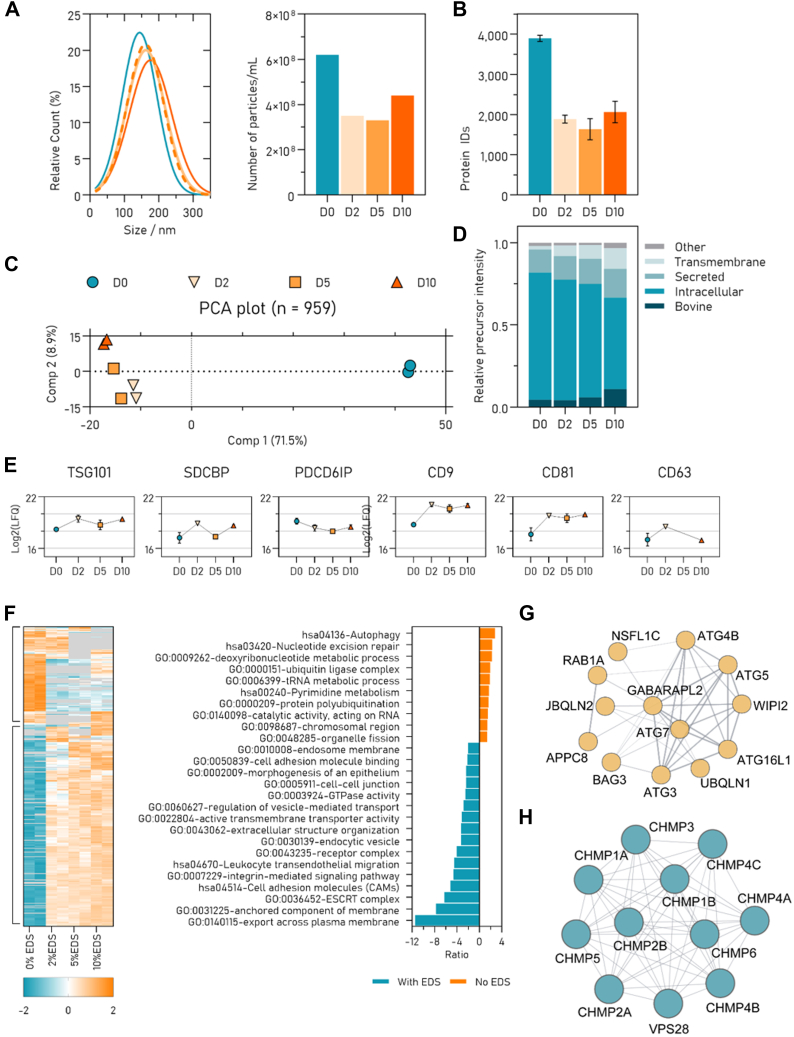
**Comparison between the EV proteomes isolated from MCF7 cells cultured in different percentages of EV-depleted FBS (EDS) reveals drastic changes in protein heterogeneity and abundances**. *A*, NTA analysis of recovered EV particles. *B*, number of protein identifications between the conditions. *C*, PCA plot indicating large variation between the relative abundances of proteins identified in serum-starved (D0) and serum-supplemented conditions (D2, D5, and D10). *D*, plot of the localization of the identified proteins. *E*, profile plot of six commonly used EV markers. *F*, heatmap of the significantly differential abundant proteins between the different conditions and the associated enriched GO terms for serum-starved (*orange*) and serum-supplemented (*teal*). Detail of ATG related proteins in the serum-starved condition (*G*) and the ESCRT associated proteins in the serum-supplemented conditions (*H*). EDS, EV-depleted FBS; D0, D2, D5, and D10, DMEM supplemented with 0%, 2%, 5%, and 10% EDS. DMEM, Dulbecco's modified Eagle's medium; EDS, EV-depleted FBS; ESCRT, endosomal sorting complexes required for transport; EV, extracellular vesicle; FBS, fetal bovine serum; GO, gene ontology; NTA, nanoparticle tracking analysis; PCA, principal component analysis.

We continued investigating the use of FAEVEr by exploring the effect of starvation on the EV proteome by isolating EVs from the CM of MCF7 cells grown in DMEM supplemented with 0%, 2%, 5%, or 10% EDS (D0, D2, D5, and D10, respectively). The FAEVEr retained EVs were purified with wash buffer containing 5% Tween-20 and either recovered for NTA (Fig. 5*A*) or lysed for LC-MS/MS analysis. The LC-MS/MS generated data immediately hinted toward an important difference by protein content and quantity between serum-deprived and EDS containing conditions as indicated in the number of identifications (Fig. 5*B*) and the principal component analysis plot (Fig. 5*C*). Over 5191 proteins were identified in total, from which 4887 were human and 304 were bovine. From the human proteins, 3610 were found in duplicate in at least one experimental setup and were included for quantitative comparison of the EV proteomes. Interestingly, we found that only 959 proteins were identified across all samples and that over 1100 proteins were uniquely identified in D0. In addition, we found that the relative abundance of transmembrane proteins gradually increased with increasing levels of EDS, whereas levels of intracellular proteins decreased (Fig. 5*D*). EV markers were abundantly picked-up across the individual samples (Fig. 5*E*). Proteins were then quantitatively compared (multiple sample testing) and hierarchical clustered (Fig. 5*F*) which lead to the conclusion that 333 proteins were significantly differential abundant over two main clusters, between the serum depleted and serum containing conditions. These two clusters were used for GO analysis using the Webgestalt v2017 online tool (67). Note that the D0 unique protein list was joined with the proteins that were significantly more abundant in the serum-depleted condition. This GO analysis revealed that EVs isolated from MCF7 cells cultured in serum-depleted conditions for only 24 h are enriched in proteins involved in metabolism, RNA and DNA binding, and autophagy, including organelle fission. Indeed, serum deprived cells are known to rapidly adjust the cellular proteome (Supplemental Fig. S7) and cells are known to upregulate autophagy related (ATG) proteins to replenish essential building blocks and release non-ESCRT dependent exosomes through the formation of autophagosomes (85), which can fuse with endosomes to form amphisomes, similar to MVBs. In addition, the ATG5-ATG16L complex (86, 87) influences the EV secretion through dissociation of V-ATPases, thereby preventing MVB acidification and its subsequent lysosomal degradation (88, 89). Ultimately, this promotes MVB fusion with the PM and secretion of the ILVs as exosomes. We found that multiple key ATG proteins and contributors of the phagosome biogenesis are high abundant in the EVs isolated from the serum-starved MCF7 cells yet nearly completely absent in the EVs isolated from the serum-containing conditions (Fig. 5, *G* and *H*). These findings are in contrast to the serum-containing conditions, where GO terms associated with (trans)membrane proteins (including transmembrane transport, cell-cell adhesion, integrins, and vesicle-mediated transport) are enriched as well as proteins associated with the ESCRT complex.

### Isolation of EVs from Complex Biofluids

We further evaluated if the FAEVEr workflow allows the efficient enrichment of EVs from more complex biofluids. Biofluids such as plasma and urine are a readily available source of biomarkers on circulating EVs and offer practical advantages over tissue biopsies. Unfortunately, EV proteome analysis using such liquid biopsies is hampered by the high abundance of contaminating proteins (*e.g.* albumin and uromodulin in plasma and urine, respectively) and the overall low EV concentration.

**Fig. 6:**
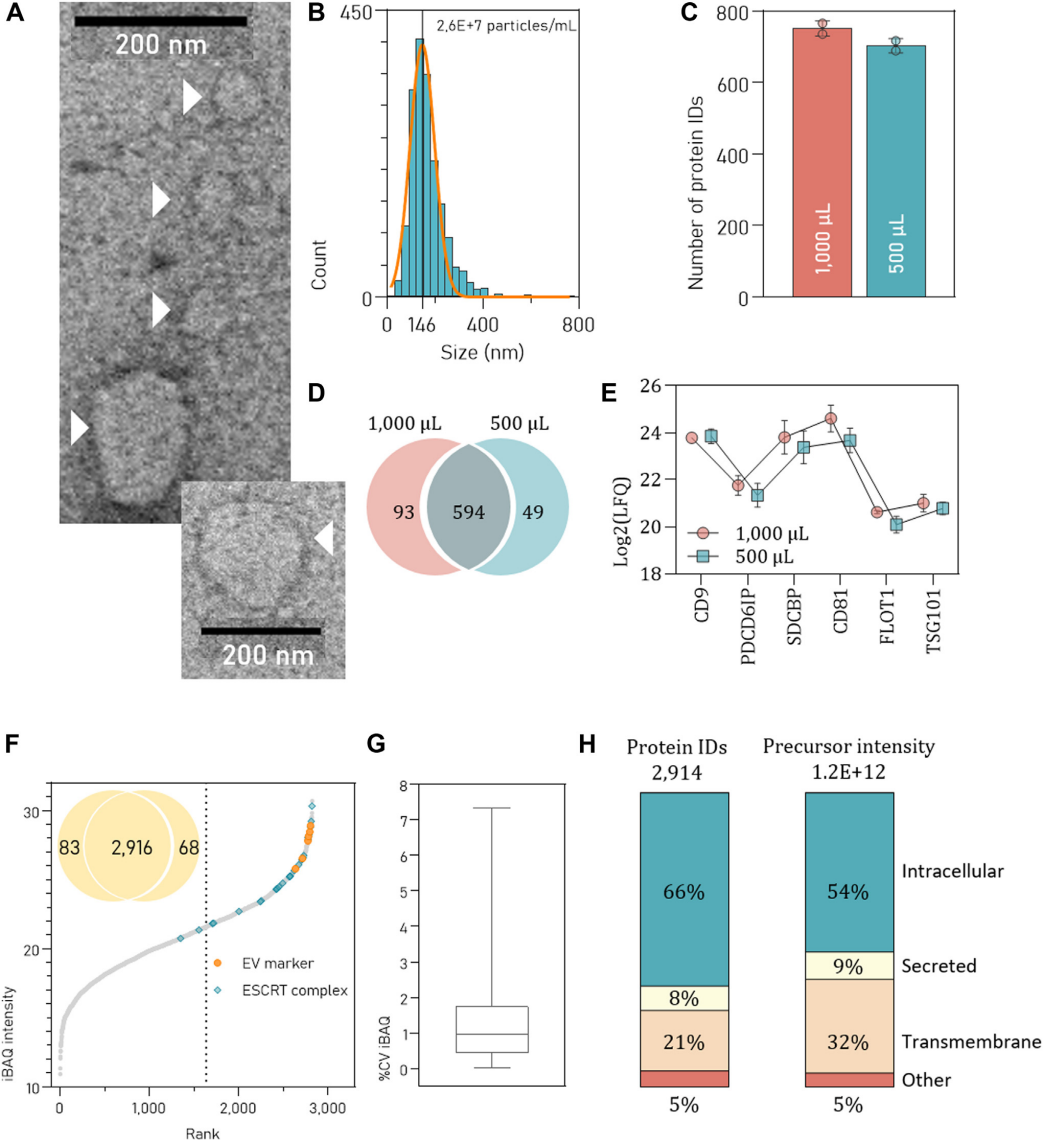
**Overview of the EV proteomes isolated from human plasma and urine**. (*A*) TEM and NTA (*B*) characterization of EVs from plasma recovered from the 300 kDa MWCO filter after finalizing the FAEVEr protocol, including 5% Tween-20 washes. *C*, number of protein identifications and (*D*) overlap of proteins that were identified in all replicates (n = 4). *E*, overview of the relative abundance (LFQ intensities) of six commonly used EV markers. *F*, number of protein identifications of EVs isolated from urine in technical duplicate and iBAQ-ranked proteins with EV markers and ESCRT-associated proteins highlighted in *orange* and *teal*, respectively. *G*, coefficient of variance of the iBAQ values between the technical replicates. *H*, pie charts depicting the number of intracellular, TM, and secreted proteins and the corresponding precursor intensities. ESCRT, endosomal sorting complexes required for transport; EV, extracellular vesicle; FAEVEr, filter-aided extracellular vesicle enrichment; LFQ, label-free quantification; NTA, nanoparticle tracking analysis; TEM, transmission electron microscopy; TM, transmembrane.

We first applied FAEVEr on plasma samples in technical duplicates using 1000 μl and 500 μl of plasma as input and characterized the retained material by NTA and TEM (Fig. 6, *A* and *B*) before analyzing the EV lysate by LC-MS/MS. Nearly 600 proteins were identified across all samples (n = 4, Fig. 6, *C* and *D*), including six general EV markers (SDCBP, TSG101, PDCD6IP, CD9, FLOT1 and CD81, Fig. 6*E*). A GO analysis (GO cellular component, GO molecular function, and GO cellular component) indicated that proteins associated with EC exosome (GO:0070062) were highly enriched in our dataset (n = 369; -log10 (FDR) = 210) together with other EV associated terms including MVB assembly (GO:0036258), vesicle-mediated transport (GO:0060627) and intrinsic component of the PM (GO:0031226). A complete overview of the significantly enriched GO annotations is provided in Supplemental Fig. S8*A*.

In addition, EVs from a urine sample were isolated in technical duplicate and FAEVEr’s reproducibility and enrichment efficiency were evaluated. Between the duplicates, 97% of the identified proteins overlapped and multiple proteins associated with EV biogenesis were highly abundant, as well as six common EV protein markers (SDCBP, TSG101, PDCD6IP, CD9, CD63, and CD81, Fig. 6*F*). In addition, the (%CV between the absolute quantification values (iBAQ values) of the individual proteins was low (median 0.95%, Fig. 6*G*). Furthermore, approximately 20% of the identified proteins were transmembrane proteins, occupying almost one-third of the spectral space (Fig. 6*H*). Secreted proteins were found in relatively low amounts (<10%), with albumin and uromodulin accounting for 2.5% and 1.3% of the total intensity, respectively, when compared to whole urine proteomics using the same digestion method (S-Trap), where albumin and uromodulin account for 33% and 4% of the total intensity (90), respectively, pointing to efficient removal of such proteins during the filter washing steps. We further evaluated our data with healthy control groups from recently published datasets. Hadisurya *et al.* studied urine EVs aimed at identifying a biomarker panel for Parkinson’s disease (91). Across 21 control patients, 3006 proteins were identified after isolating EVs using the EVTrap approach, compared to 2957 identifications from FAEVEr from which 1689 overlapped. Quantitative comparison of the proteins that were identified across all samples (Supplemental Fig. S8*B*) revealed that FAEVEr-enriched proteins were associated with (among others) MVB sorting, GTPases and TM transporter activity, whereas the EVTrap dataset was enriched in terms including peptidase/hydrolase activity, collagen-containing extracellular matrix and blood microparticle (Supplemental Fig. S8*C*). In addition, our FAEVEr dataset was compared with results from the study conducted by Khoo *et al.* (92), In that study, EVs were isolated from urine by UC (150,000*g*) to identify differences in the proteome between men with prostate cancer and respective controls. Here, we found that 75% of the identified proteins of the control group overlapped with our dataset (Supplemental Fig. S8*D*). We then compared the absolute levels of the proteins between the two approaches by comparing iBAQ values, and visualized the results in a density scatter plot. We found that the overall proteome correlated well (Pearson correlation = 0.67, Supplemental Fig. S8*E*) and that proteins associated with the ESCRT machinery and the general EV protein markers (CD9, CD81, CD63, SDCBP, TSG101, and PDCD6IP) were found at similar (high) levels in both EV enrichment strategies.

## Conclusion and Discussion

Despite the potential of EVs for clinical applications such as being a source of circulating biomarkers or therapeutic shuttles, their efficient isolation and purification from complex biofluids in a cost and time efficient manner remains a major bottleneck. Here, we propose FAEVEr as an EV enrichment strategy that uses 300 kDa MWCO filtration in combination with a washing buffer containing up to 5% Tween-20 toward efficient removal of non-EV proteins.

In this study, rEVs (50, 51)) were used to initially establish and optimize our strategy. We made use of the overexpression of a luminal Gag-eGFP construct to evaluate the rEV integrity during the enrichment and purification and concluded that during FAEVEr the rEV particles indeed remain intact. In addition, we concluded that the EVs can be recovered from the filter membrane for further evaluation using common tools for EV characterization such as NTA and SEM and TEM. In addition, the rEV proteome was quantitatively recovered by immediately lysing the filter-retained EVs for LC-MS/MS using a high concentration of SDS.

We benchmarked our approach against UC, a widespread implemented method for EV enrichment. We observed a drastic decrease in the relative variance (%CV) between the number of identifications on both the protein, peptide, and peptide-spectral match level between UC and FAEVEr. This also indicates a higher reproducibility between replicates, which in turn leads to improved data completeness (fewer missing values) and, consequently, improved quantifications. We hypothesize that this improvement is partly due to the absence of intermediate pipetting steps that remove the supernatant after UC, which potentially leads to incomplete removal or unintended perturbations of the EV pellet. Instead, FAEVEr establishes a clear separation between the EV-enriched retentate and the EV-depleted filtrate, which can be simply decanted. We noticed a substantial difference in sample preparation time, with UC requiring at least two rounds of centrifugation, totaling to 3 hours of centrifugation time, whereas FAEVEr was finished within the hour, including centrifugation steps and hands-on time.

We continued to improve the EV purity by implementing Tween-20 in wash buffers as this detergent was previously shown not to lyse EVs, even in relatively high concentrations (75), and it is an established supplement to reduce nonspecific protein-membrane interactions, for example in Western blotting. We provide insights in the gradual loss of bovine serum proteins and secreted proteins, to which we refer here as non-EV proteins. This improved removal of unwanted proteins is of high importance for LC-MS/MS based bottom-up proteomics, as precursors from such high abundant proteins (*e.g.* albumin) dominate the spectral space, outcompeting relevant EV precursors. We observed that FAEVEr in combination with 5% Tween-20 resulted in optimal results but the same profound effect was absent in combination with UC. Indeed, a number of proteins associated with (among others) transcription and translation, proteasomal degradation, and metabolic activities were more prominent in all UC samples as well as in the FAEVEr setup without Tween-20 supplemented to the wash buffers. In contrast, the Tween-20 washed EV proteomes following the FAEVEr protocol were enriched in (trans)membrane proteins and in proteins from the ESCRT machinery. We hypothesize that during cell culture inevitable cell lysis occurs during which cytosolic proteins are released and copurified during UC. These insights raise the question whether or not such proteins are effectively encapsulated in EVs or if they form non-specific protein interactions with the EV membrane. However, additional studies are required to confirm this.

In EV research, many experiments exclude serum supplements in cell culture media to repulse xeno-proteins and omit the suppressive competitive effect during LC-MS/MS analysis and the potential biological influence on the EV proteomics outcome. We first investigated on the difference between regular FBS and EDS on the LC-MS/MS level, from which we concluded that EV markers and biogenesis related proteins are virtually absent in EDS, implying that we do not contaminate our EV preparation unintendedly with bovine EV particles. We further elaborate that the depletion of serum during cell culture, even for a limited period of time (*e.g.* 24 h), has a dramatic effect on the EV proteome of MCF7 cells. In line with Leidal *et al.* (85) we observed an increase in protein heterogeneity associated with RNA binding, biosynthesis, cell cycle, and autophagy, pointing toward an alternative biogenesis of EV particles through the formation of amphisomes. These findings are underlined by the unique presence of several ATG proteins in the EV preparation of serum-starved MCF7 cells. In contrast to this, the EV proteome from MCF7 cells supplemented with EV-depleted FBS, contained higher levels of proteins associated with transmembrane transport, cell-cell adhesion and the ESCRT-machinery were more abundant. We feel that the choice to add or exclude EDS during cell culture for EV research is an important consideration when testing a preconceived hypothesis as it may bias the interpretation of the EV (proteomics) data (93, 94).

Lastly, we explored FAEVEr for the enrichment of EVs from human plasma and urine, both readily accessible sources for biomarker discovery. Using plasma, we compared 1 ml and 500 μl sample input and found that, in both cases, comparable numbers of proteins were identified with a high overlap, without filter clogging or irreversible fouling, a previously major concern in dead-end UF of plasma. Apart from the consistent identification of EV markers, the commonly identified proteins were highly enriched in EV associated proteins, including GO terms such as EC exosome (GO:0070062), MVB assembly (GO:0036258), vesicle-mediated transport (GO:0060627) and intrinsic component of the PM (GO:0031226). Furthermore, using TEM and NTA characterization, we showed that the plasma-derived EVs remained intact after washing with 5% Tween-20. For urine, EV isolation appeared to be successful as well, with consistent numbers of identified proteins between technical replicates, with a relative high abundance of TM proteins (20%) and associated precursor intensities (32%). Following comparison with two recently published datasets, we found that FAEVEr resulted in comparable EV proteomes with a good overall correlation of absolute abundances (iBAQ values), including the EV markers and ESCRT associated proteins (92). Furthermore, in comparison with Hadisurya *et al.* (91), we found that our dataset was enriched in GO terms associated with, among others, MVB sorting, GTPase activity and TM transport activity, yet depleted of proteins associated with GO terms such as blood micro particle, collagen-containing extracellular matrix and platelet alpha-granules. From these results, we conclude that FAEVEr results in proteome results comparable to EVTrap (91) and UC (92) with a high abundance of EV markers, TM proteins and proteins associated with EV biogenesis. However, future studies will have to challenge the applicability of the FAEVEr approach using large cohorts of biological samples to validate FAEVEr’s possible clinical value of detecting quantitative differences between EV proteomes within patient cohorts, especially regarding the expected lower input samples.

In conclusion, FAEVEr using 300 kDa MWCO PES membrane filters has been validated to retain intact EVs from various sources, including CM as well as complex biofluids including serum, plasma, and urine. Despite the coenrichment of same-size particles, the EV proteome reaches LC-MS/MS compatible purity, even at 10% EDS, by including 5% Tween-20 in the washing buffer, which greatly diminishes membrane fouling. We showed that Tween-20 does not affect the EV integrity during UF at moderate speed, even when used at relative high percentages (up to 5%), although it proved difficult to recuperate filter-retained EVs quantitatively. In addition, UF is conducted using standard lab equipment, in a very reasonable timeframe and offers the possibility toward parallelization.

## Data Availability

The mass spectrometry proteomics data have been deposited to the ProteomeXchange Consortium *via* the PRIDE partner repository with the dataset identifiers PXD051938 (comparison FAEVEr with UC), PXD051955 (comparison MCF7 proteome of EVs and cells under starving conditions), PXD051956 (comparison FBS with EDS), and PXD056479 (exploratory study of EVs from human plasma and urine). All required details concerning the enrichment of extracellular vesicles were submitted to EV-TRACK (EV240045). All spectra were submitted to Panorama (Skyline).

For review:

PXD051938 Username: reviewer_pxd051938@ebi.ac.uk; Password: vdJm9iCb

PXD051955 Username: reviewer_pxd051955@ebi.ac.uk; Password: GjVmeCax

PXD051956 Username: reviewer_pxd051956@ebi.ac.uk; Password: RpPtUmnd

PXD056479 Username: reviewer_pxd056479@ebi.ac.uk; Password: qRGonf3xdEEr

Panorama (Skyline, https://panoramaweb.org/ZHReHg.url); Password: o5i6&xy1EJAFˆ9 Username: panorama+reviewer289@proteinms.net

## Consent for publications

All co-authors have consented to publication.

## Supplemental Data

This article contains supplemental data.

## Conflict of interest

The authors declare no competing interests.

## Supplementary Data

Suppl Figure 1

Suppl Figure 2

Suppl Figure 3

Suppl Figure 4

Suppl Figure 5

Suppl Figure 6

Suppl Figure 7

Suppl Figure 8

Suppl data A

Suppl data B

Suppl data C

Suppl data D

Suppl data E

Suppl data F

Supplemental Figure Caption
