# Supplementary figures and images for: Filter-Aided Extracellular Vesicle Enrichment (FAEVEr) for Proteomics
Source: Mol Cell Proteomics. 2025 Jan 21;24(2):100907. doi: 10.1016/j.mcpro.2025.100907 (PMC11872570; doi:10.1016/j.mcpro.2025.100907)

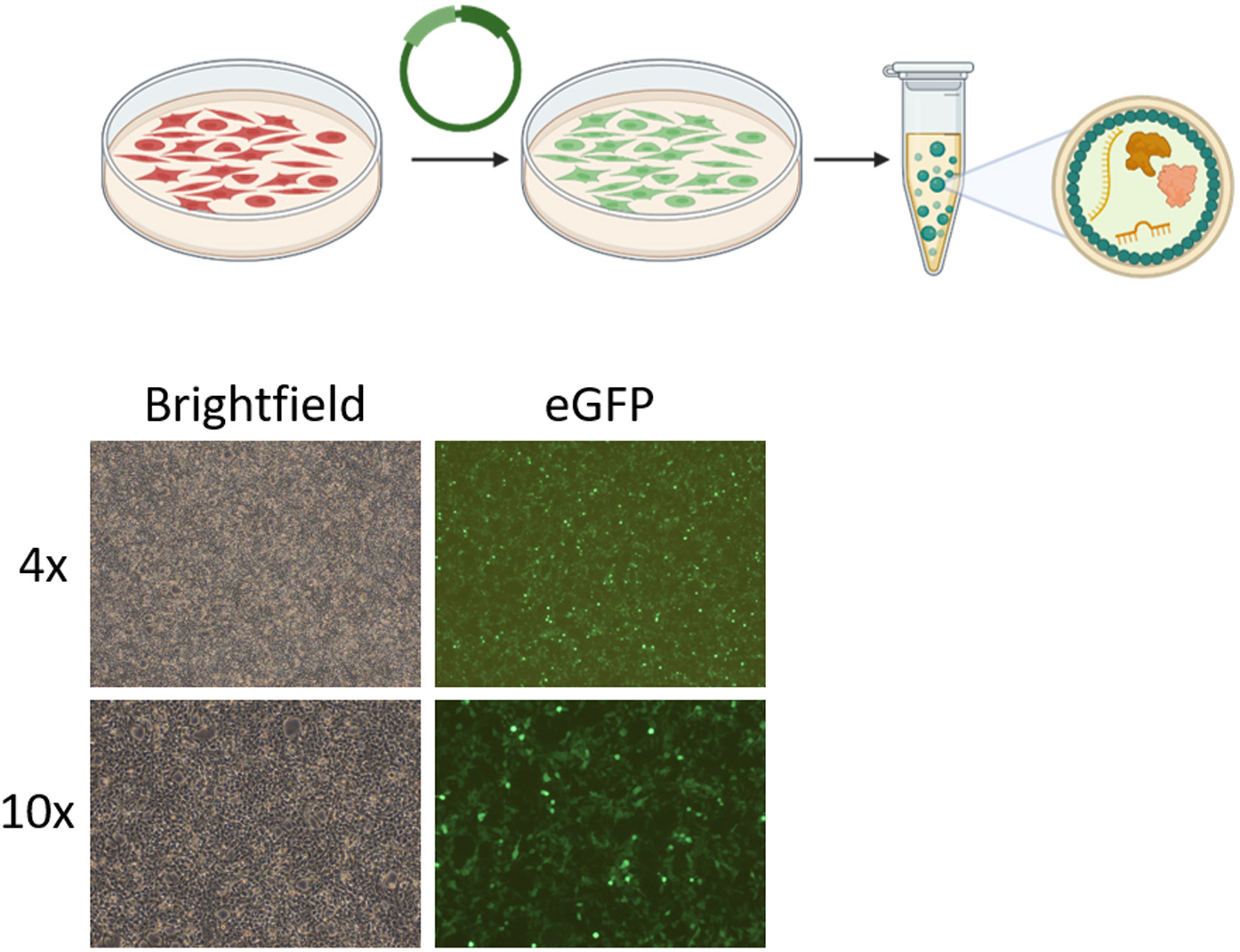

Supplement: Suppl Figure 1 [file figs1.jpg]

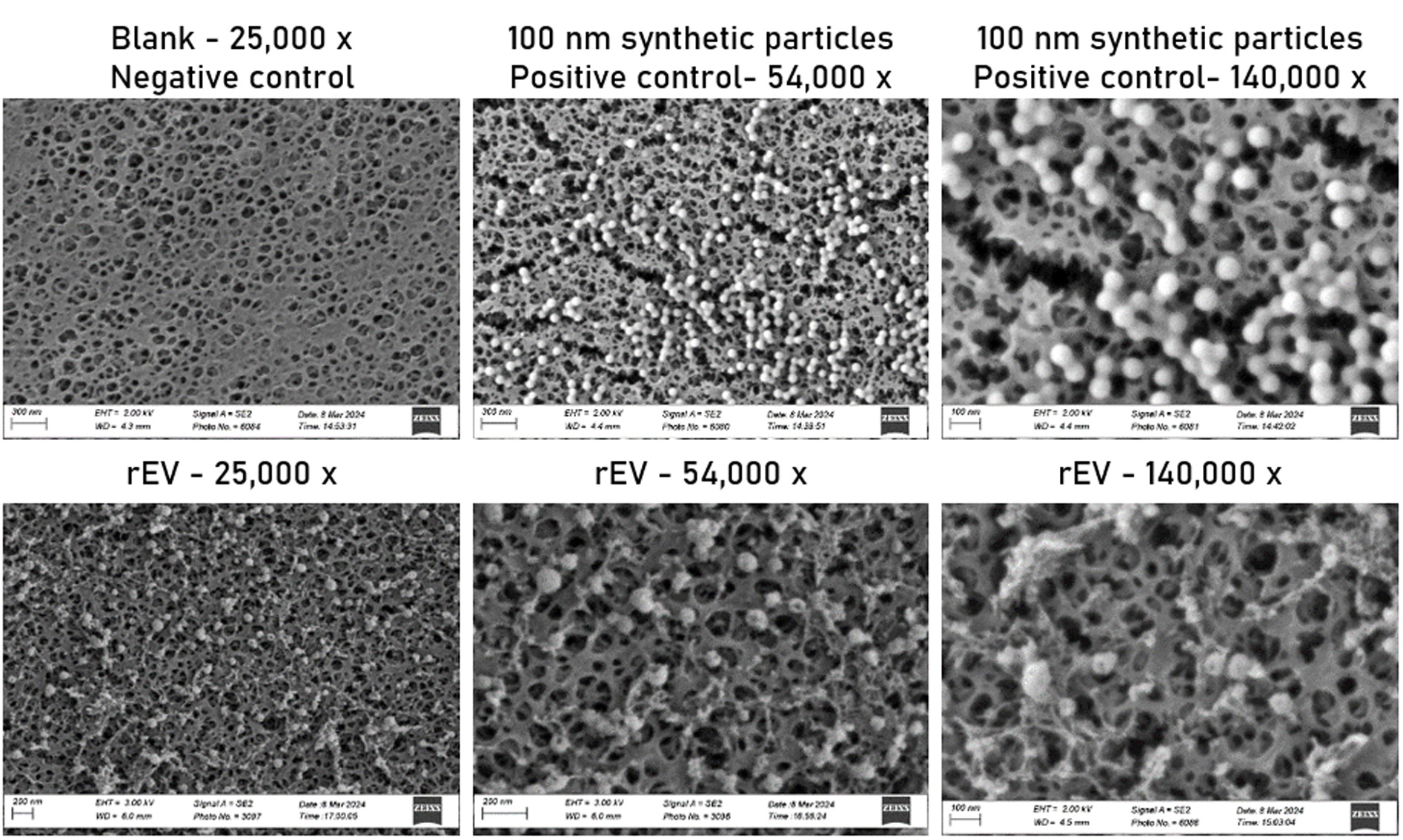

Supplement: Suppl Figure 2 [file figs2.jpg]

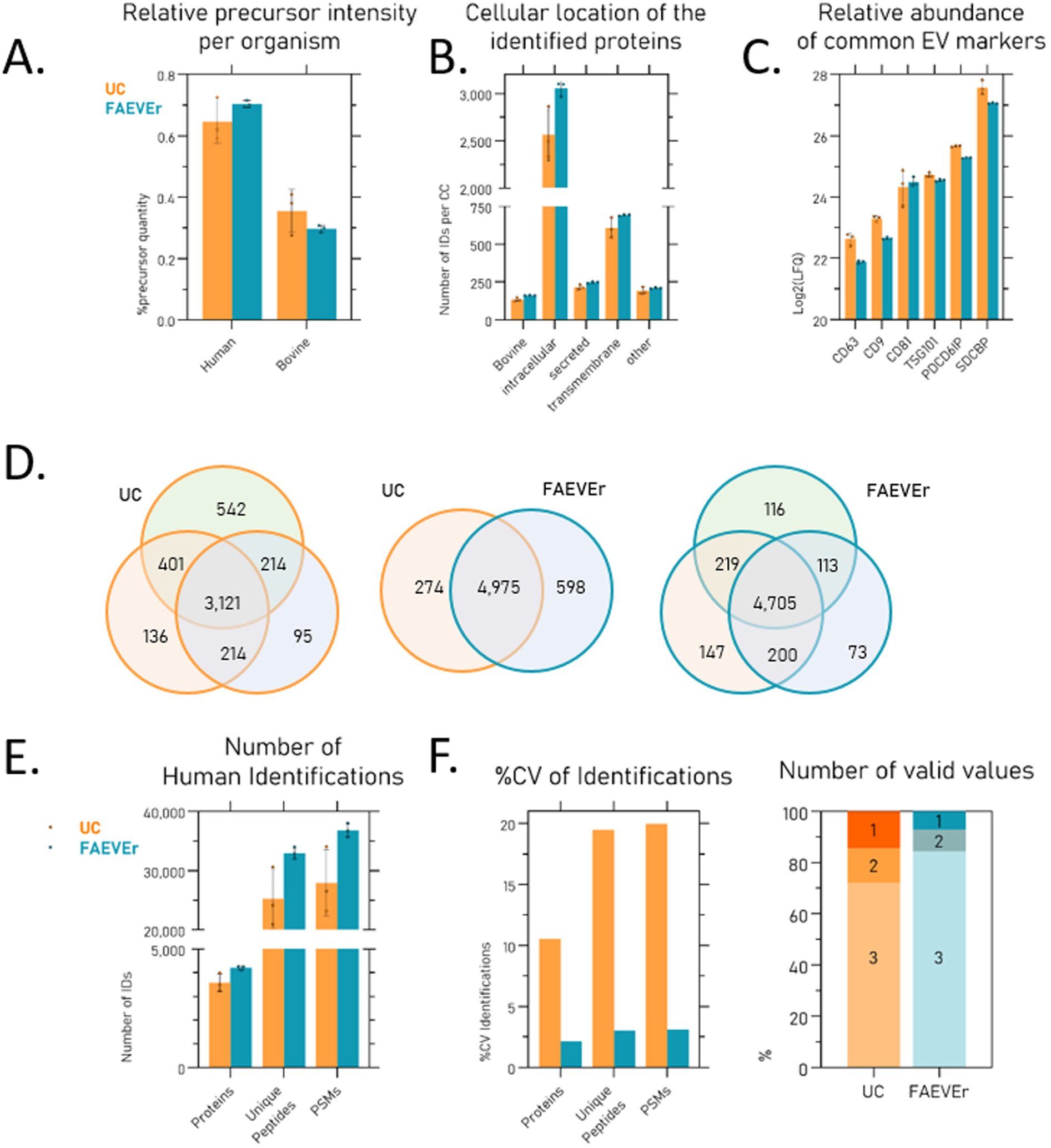

Supplement: Suppl Figure 3 [file figs3.jpg]

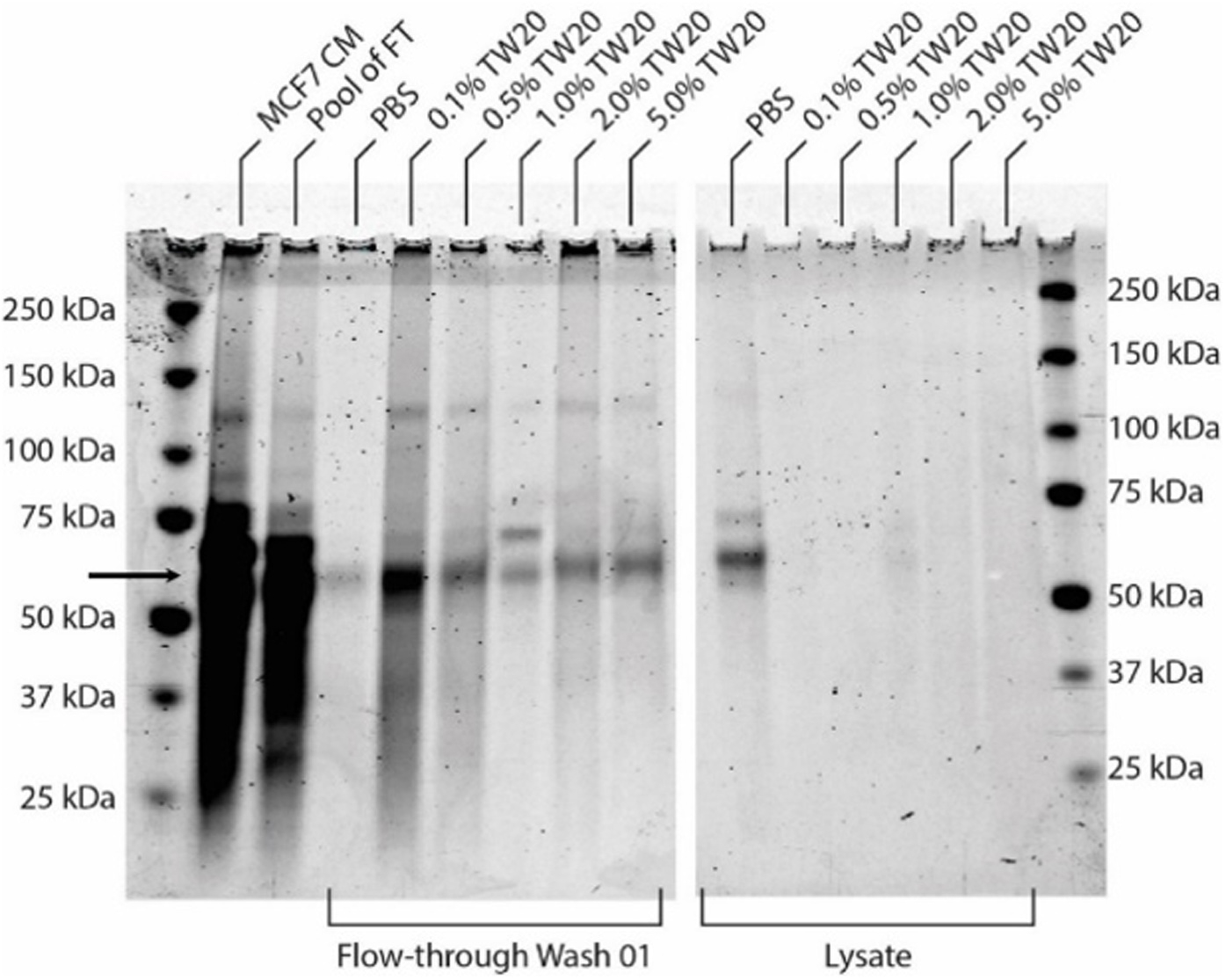

Supplement: Suppl Figure 4 [file figs4.jpg]

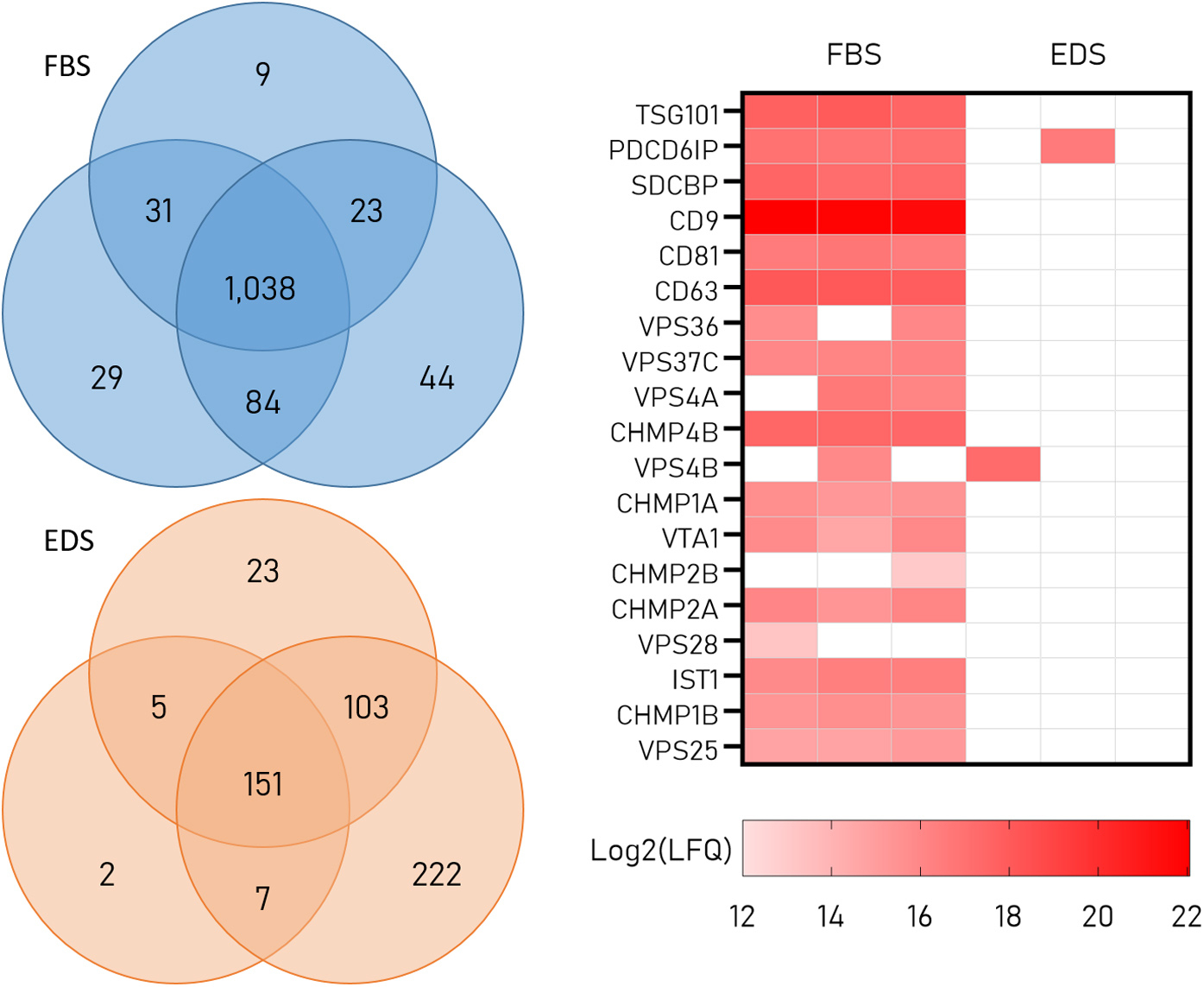

Supplement: Suppl Figure 5 [file figs5.jpg]

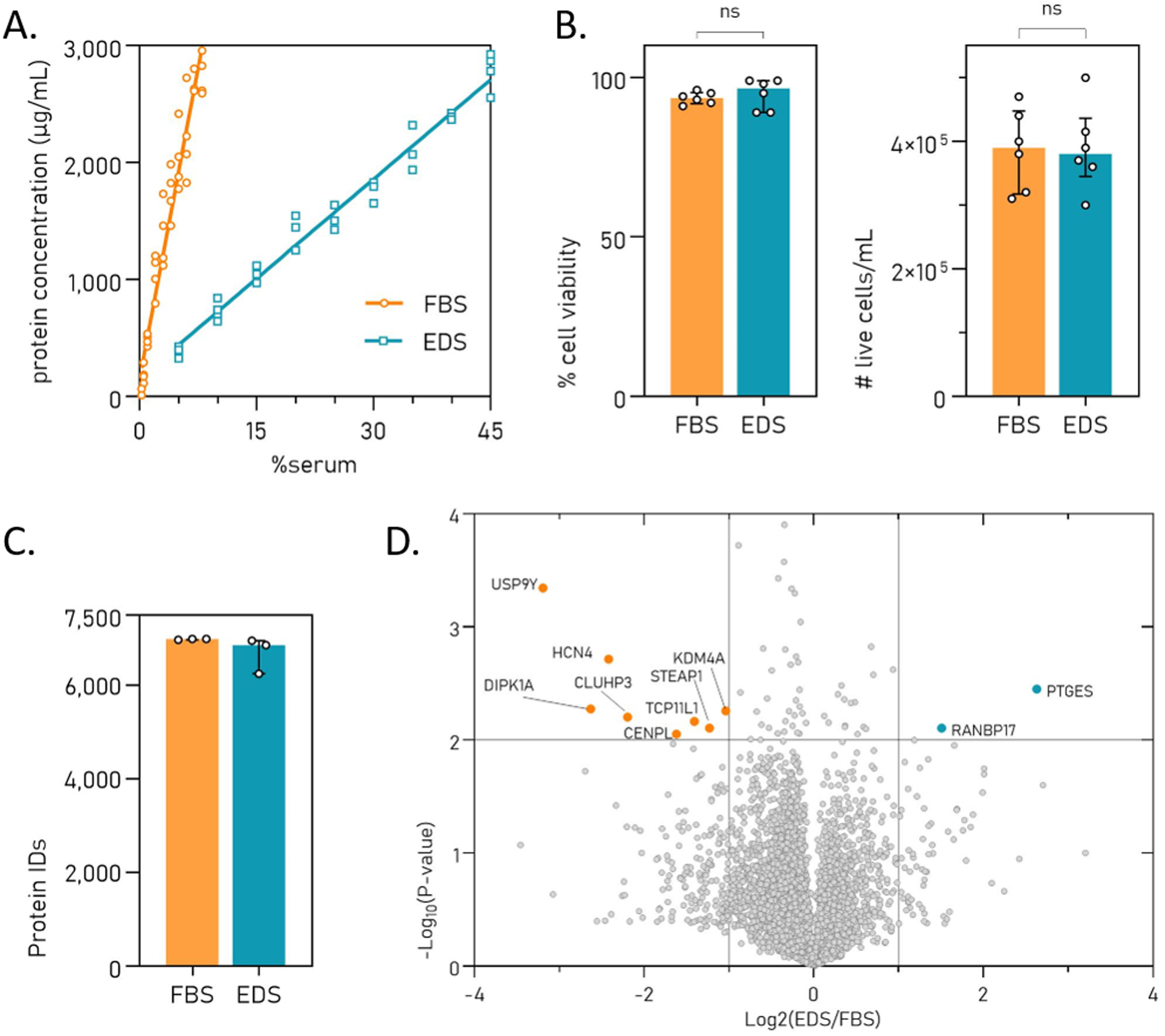

Supplement: Suppl Figure 6 [file figs6.jpg]

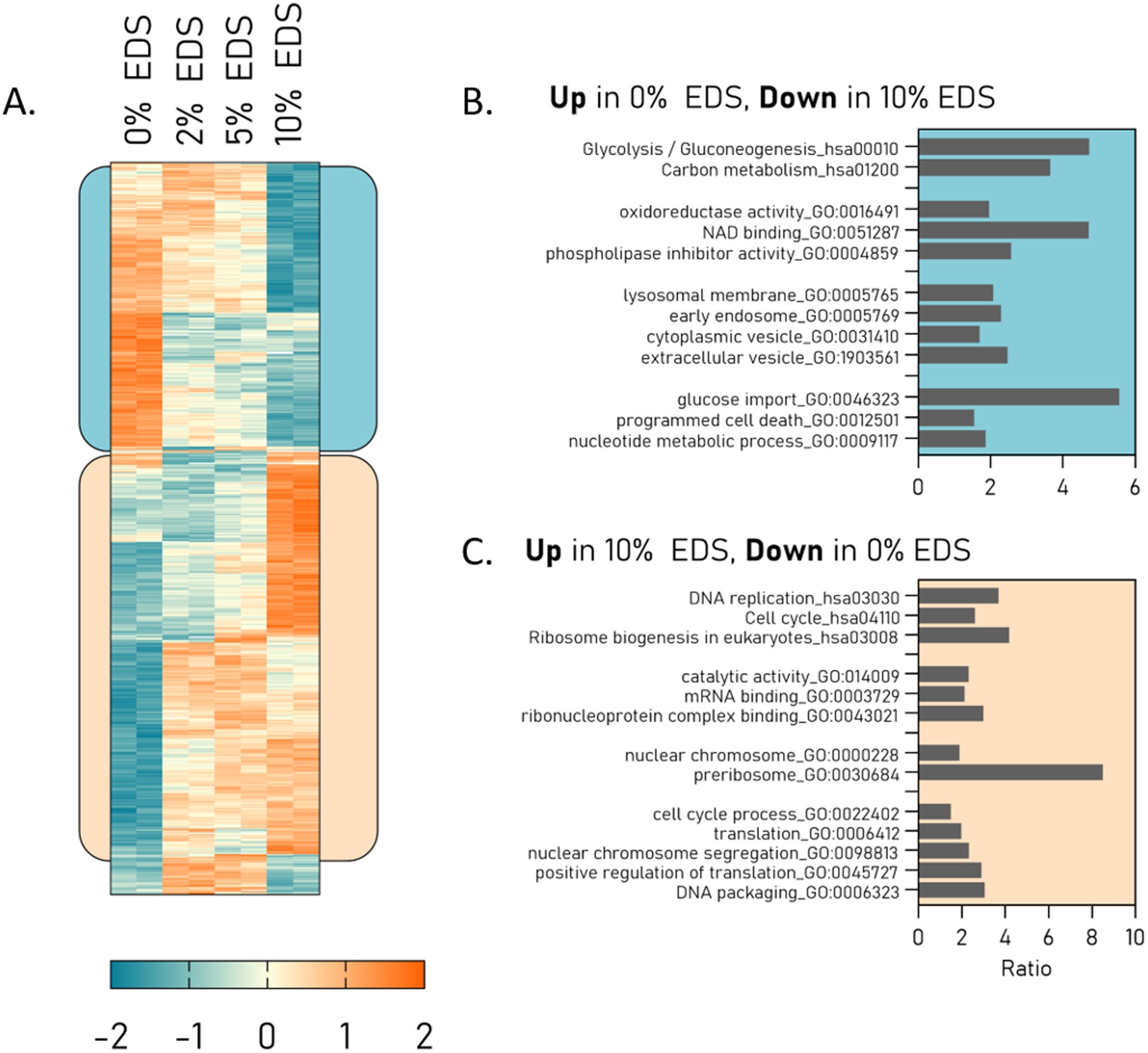

Supplement: Suppl Figure 7 [file figs7.jpg]

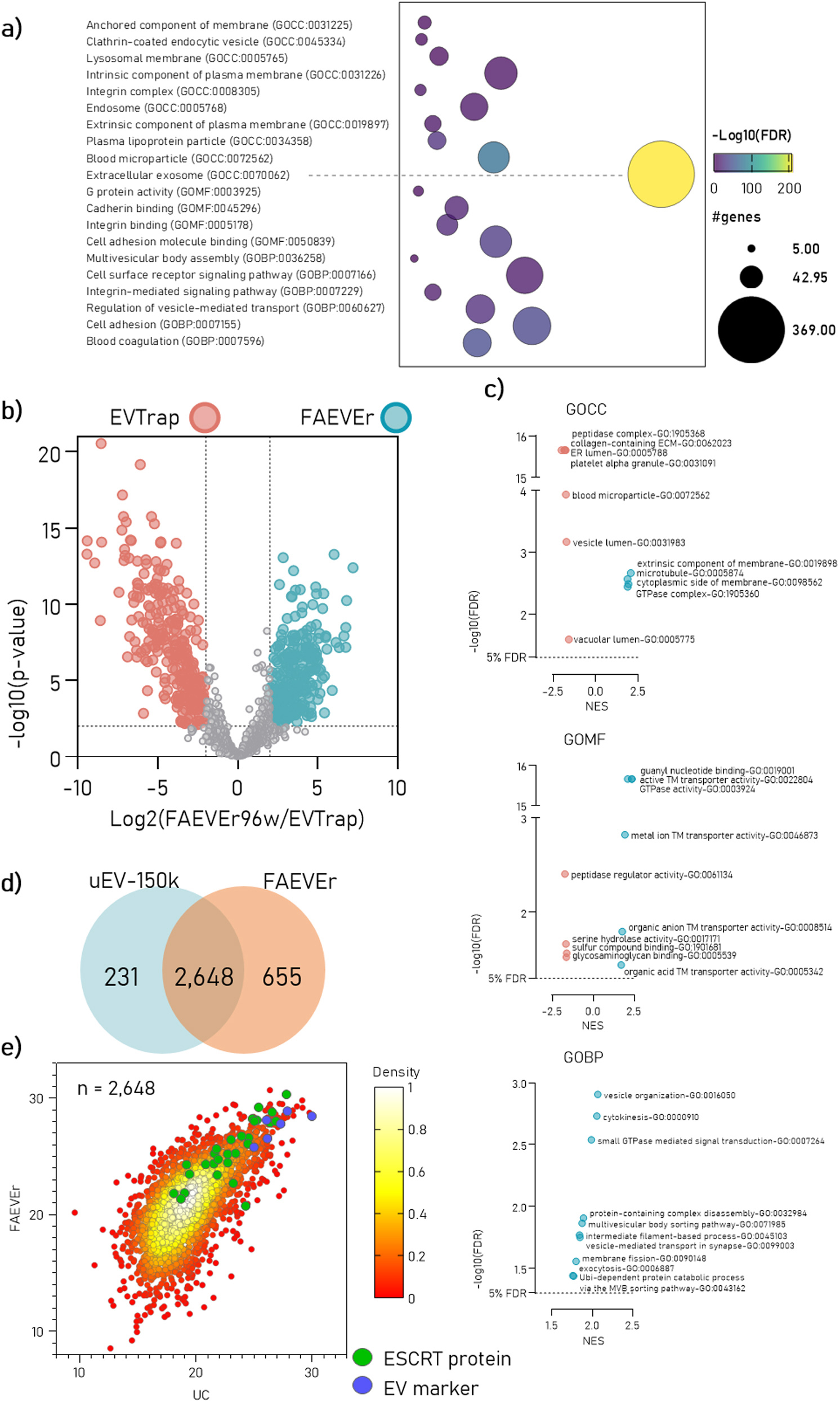

Supplement: Suppl Figure 8 [file figs8.jpg]
