# Supplementary material for: Filter-Aided Extracellular Vesicle Enrichment (FAEVEr) for Proteomics
Source: Mol Cell Proteomics. 2025 Jan 21;24(2):100907. doi: 10.1016/j.mcpro.2025.100907 (PMC11872570; doi:10.1016/j.mcpro.2025.100907)
Supplement: Supplemental Figure Caption [file mmc7.docx]

Supplementary Figure 1: Schematic overview of the expression of GAG-eGFP in HEK293T cells. Top: HEK293T cells are transfected with a Gag-eGFP construct, encoding for a Gag-eGFP fusion protein that multimerizes at the endosomal and plasma membrane, inducing the formation of virus-like particles packed with eGFP. Bottom: brightfield and eGFP pictures of HEK293T cells after transfection, prior to the isolation of conditioned medium.

Supplementary Figure 2: Scanning electron microscopy of 300 kDa MWCO filters used in FAEVEr. Blank: empty filter membrane with clear visualization of the 300 kDa MWCO filter membrane. Synthetic particles: homogeneous synthetic particles used for NTA calibration were retained on the filter after completing the FAEVEr protocol and serve as a positive control. rEV: retained rEV particles after completing the FAEVEr protocol with a distinct delineation of the EV particles against the 300 kDa MWCO membrane.

Figure 3: Overview of the comparison between FAEVEr and ultracentrifugation. A) Comparison of the relative abundance of precursor material from bovine or human origin. B) Distribution of the protein identifications across the cellular localizations of the parental cell. C) High relative abundance of six molecular EV markers. D) Overlap of protein identifications between UC and FAEVEr. E) Total number of identified human proteins, identified unique peptides and peptide-spectral matches from these proteins between UC and FAEVEr. F) Decreased relative variance (%CV) and fewer missing values are observed with FAEVEr compared to UC. UC: ultracentrifugation (orange), FAEVEr: Filter-aided EV enrichment (teal), CC: cellular compartment, LFQ: label-free quantification intensity, PSM: peptide-spectral match, %CV: coefficient of variance.

Supplementary Figure 4: SDS-PAGE with Coomassie staining indicates quantitative removal of serum proteins with FAEVEr when TWEEN-20 is supplemented to the filter, indicating decreased membrane fouling. In the PBS condition (no TWEEN-20) a faint band is observed at 65 kDa, indicating that only a fraction of the bovine albumin is removed by the wash steps. This stands in contrast to washing with buffers that are supplemented with of TWEEN-20, where more intense bands are observed in the washes. Additionally, the lysate of PBS contains observable more albumin material, further indicating that PBS alone fails to remove the bulk of contaminants contrary to TWEEN-20 were the lysate does not contain such strong signal.

Supplementary Figure 5: Comparison of the identified proteins in standard fetal bovine serum (FBS) and EV-depleted FBS (EDS). A) After completing FAEVEr and subjecting the lysate to LC-MS/MS analysis, we observed a high number of identifications in FBS but not in EDS, indicating a higher protein heterogeneity due to the presence of EV particles. B) EV markers or biogenesis related proteins are virtually absent in EDS, yet highly abundant in FBS.

Supplementary Figure 6: Culturing MCF7 cells in 10% standard fetal bovine serum (FBS) or 10% EV-depleted FBS (EDS) for three days, has no huge effect on the MCF7 cellular proteome. A) Due to the EV depletion protocol, EDS contains approximately six times less protein material. B) No significant changes in cell viability or cell count were observed. C) In both conditions, we identified over 7,000 proteins from which only 10 were significantly differentially regulated (D).

Supplementary Figure 7: The cellular proteome of MCF7 cells cultured at different percentages of EV-depleted FBS (EDS) changes between serum-starved (0% EDS for 24 h) and optimal conditions (10% EDS for 48 h). A) Heatmap depicting the differentially regulated proteins between the different conditions from which we further investigated two major clusters to evaluate the contrast between 0% EDS and 10% EDS. Gene ontology (GO) analysis of the proteins up-regulated in the serum-starved conditions (B) and optimal conditions (C).

Supplementary Figure 8: The proteome of EVs isolated from biological fluids. The proteome of EVs isolated from biological fluids. (a) Gene ontology analysis of the EV proteome isolated from plasma reveals enrichment of proteins associated with extracellular exosomes and related terms. (b) Volcano plot depicting significantly differentially abundant proteins after EV isolation by EVTrap (left, red) and FAEVEr (right, teal) with the associated significantly enriched GO terms. (c) Overlap of the protein identifications between FAEVEr and UC (150,000 x g) and correlation (Pearson correlation = 0.67) between the individual iBAQ values with EV markers and EV biogenesis related proteins indicated in blue and green, respectively.
